# Rapid Detection of Tetracycline Residues in Milk Using Colorimetric Sensor Based on Polymethylmethacrylate (PMMA) and Polystyrene (PS) Polymers

**DOI:** 10.1155/jamc/5412774

**Published:** 2026-05-20

**Authors:** Rimadani Pratiwi, Putri Nur Azizah, Aliya Nur Hasanah

**Affiliations:** ^1^ Department of Pharmaceutical Analysis and Medicinal Chemistry, Faculty of Pharmacy, Universitas Padjadjaran, Sumedang, 45363, Indonesia, unpad.ac.id

**Keywords:** colorimetric sensor, indicator strip, PMMA, polymer, PS, tetracycline

## Abstract

In this study, the colorimetric approach is applied on an indicator strip for tetracycline detection in milk residue. Indicator strips made from PMMA‐concentrated H_2_SO_4_ (9:1), PMMA‐Mecke (9:1), and PMMA‐Marquis (9:1) are the best compositions that can detect tetracycline within 10 s. The test results using scanning electron microscopy–energy‐dispersive X‐ray spectroscopy (SEM‐EDS) and infrared spectrophotometer showed that the reagent had entered the PMMA‐based indicator strip. The limit of quantification (LOQ) and limit of detection (LOD) were 11.64 ppm and 3.49 ppm, respectively. The indicator strip has high selectivity toward tetracycline and is stable for 60 days at room temperature in a desiccator. The indicator strip detects tetracycline in milk samples with an accuracy of 83.24%–99.66%. The accuracy test results using liquid chromatography–tandem mass spectrometry (LC‐MS/MS) also observed. The indicator strip may be appropriate as an initial screening tool for high‐level tetracycline contamination; however, its utility is limited for purposes of strict regulatory compliance.

## 1. Introduction

Milk is an essential animal product for humans as it includes minerals and protein. Protein comprises amino acids, which help to create bodily tissue, grow, and replace damaged cells [1]. According to forecasts for the years 2022–2026, Indonesia’s cow milk consumption will increase by 2.07% annually [2]. The increase in milk consumption has made farmers try to improve the quality of their livestock to produce more milk. Livestock are generally susceptible to various diseases, especially in the respiratory and urinary tract, such as mastitis, usually caused by bacteria that can reduce milk production and poor meat quality [3, 4]. Farmers frequently employ tetracycline antibiotics to treat these infections because they have the ability to kill a wide range of germs, are inexpensive, are simple to use, and have mild side effects [5].

In addition to treating diseases, farmers often misuse tetracycline as daily feed or feed additives to improve milk production, protein over fat, and development by up to 10% [6, 7]. Tetracycline can enter milk because it has a hydrophobic group that is more soluble in fat [8]. Excessive use of tetracycline can cause yellow teeth, severe allergies, bacterial resistance, fetal defects, liver damage, kidney damage, and even death [9–11]. Therefore, there are regulations governing the limits of tetracycline residues allowed in food products issued by the Food and Agriculture Organization (FAO) in the Codex Alimentarius Commission (CAC), the European Union (EU), and the Indonesian National Standard (SNI) in Indonesia. The maximum residue limit (MRL) of tetracycline residues in milk required by the EU and FAO is 100 ppb [12, 13]. SNI requires 0.05 ppm as the limit of tetracycline residues in milk [14].

The methods that are widely used to detect tetracycline antibiotics in milk samples are UV–Vis spectrophotometry, high‐performance liquid chromatography (HPLC), liquid chromatography–tandem mass spectrometry (LC‐MS/MS), immunoassay, capillary electrophoresis, and electrochemistry [15–19]. Despite its high selectivity and sensitivity, the immunoassay method can produce false positives due to the autoantibody process [20]. Electrochemical methods also require special equipment that can conduct electricity and the appropriate pH so that the device detects the analyte [21]. Other methods, such as LC‐MS/MS, HPLC, capillary electrophoresis, and UV–Vis spectrophotometry, can provide accurate quantitative results, good selectivity, and high sensitivity. However, these methods require long operating times, expensive prices, and trained personnel [22].

Polymer‐based detection devices using colorimetric principles have recently gained popularity for detecting tetracycline residues in milk. Colorimetric approaches use specific reagents to interact with analytes, resulting in interpretable color changes [22]. Several studies on paper‐based detection devices, such as those conducted by Lu et al. [23], Sun et al. [24], and Teixeira & Sales [25], have demonstrated visually observable color changes without the need for specialized instruments. However, the detection time remains relatively long, typically exceeding 1 minute. Other paper‐based studies reported by Jia et al. [26], Li et al. [27], and Wu et al. [28] achieved faster detection times (≤ 1 min), but these methods require a fluorometer, limiting their applicability outside laboratory settings. Although both approaches provide good selectivity and sensitivity, there is still a need for methods that offer rapid analysis while remaining operable without specialized instrumentation. Therefore, this study focuses on developing a paper‐based sensor capable of providing rapid detection while being portable, selective, and sensitive, thereby offering a novel solution not yet achieved by previous studies and potentially improving the accessibility and effectiveness of analytical applications in the field of analytical chemistry.

Polymethylmethacrylate (PMMA)‐based polymer test strip method has been previously developed for detecting Pigment Red 53 in cosmetics [38] and dexamethasone in herbal medicine [40], and the result shows that this method is simple and applicable for onsite screening analysis with good performance. In this research, the use of these polymers for tetracycline detection in samples will be investigated. This study will be carried out to construct indicator strips from PMMA polymers and a combination of polystyrene (PS) and PMMA mixed with specific reagents, including concentrated H_2_SO_4_, Mecke, and Marquis [29]. Concentrated H_2_SO_4_, Mecke, and Marquis reagents contain strong acids; hence, the fundamental material of the selected indicator strip must be acid‐resistant. PS is a polymer that is resistant to strong acids, an essential chemical, hydrophobic, and oxidizing and reducing agents [30]. PMMA is a hydrophilic polymer resistant to dilute acids, elastic, strong, and stable at high temperatures, and provides stable color changes [31, 32]. The mixture of hydrophobic and hydrophilic polymers will increase the pore size to increase the interaction of the active side of the analyte with the reagent [33]. The reagent blending method creates the indicator strip by mixing the polymer and chemical reagents in a liquid form. It is better than solvent impregnation because the reagent interacts directly with the polymer, causing the functional groups to change, and the reagent will last longer in the pores [34]. In addition, ImageJ is used for measurement analysis. This mixture of PS and PMMA polymers is expected to create a portable indicator strip and produce fast, onsite, selective, and sensitive analysis.

## 2. Materials and Methods

### 2.1. Reagents and Materials

Analytical‐grade reagents used include ammonium hydroxide 25%, phosphoric acid, perchloric acid, selenious acid, concentrated sulfuric acid, acetonitrile, ethyl acetate, chloroform, formaldehyde 37%, magnesium sulfate, sodium hydroxide, sodium chloride, and anhydrous sodium sulfate. Meanwhile, formic acid 0.1%, oxalic acid, and methanol were used in HPLC grade. TLC silica gel plates (GF_254_) were obtained from Merck. Tetracycline hydrochloride, PS polymer, and PMMA polymer were supplied by Sigma‐Aldrich. Ampicillin injection powder was obtained from Bernofarm. Enrofloxacin and tylosin injectable solutions were sourced from Sanbe Farma and Medion Farma. Additionally, the injection powders of kanamycin sulfate and streptomycin sulfate were produced by Meiji Indonesian Pharmaceutical Industries. Raw goat and cow milk samples were taken from a West Java Province, Indonesia farm.

### 2.2. Instrumentation

The concentration of tetracycline residues was analyzed using LC‐MS/MS AB Sciex TQ 4500 with ultraperformance liquid chromatography (UPLC) Waters Acquity I‐Class using an ACQ‐BEHC18 column. The gradient pump system used the mobile phase of 0.1% formic acid in aquabidest and methanol. The flow rate used was 0.25 mL/min. An attenuated infrared (IR) total reflectance spectrophotometer (IR‐ATR Prestige 21 Shimadzu Made in Japan) was used to observe the mixing of polymers and reagents based on changes in functional groups. A scanning electron microscope (SEM JEOL JSM‐6510LA) was used to observe the shape of the pores of the indicator strip. The color intensity of the indicator strip tested with standards and samples was analyzed using the ImageJ application Version 1.8.0.

### 2.3. Sample Collection and Preparation

Samples were taken from various goat and cow farms in West Java, Indonesia, especially Bogor and Bandung. Three goat milk samples and three unpasteurized cow milk samples were sent at room temperature on the same day as the order. The method used for tetracycline extraction from milk samples is the magnesium precipitation method, which has been proven to be fast and provides a reasonably significant recovery value [35]. The collected milk samples were stored in a freezer (less than −17°C) [36]. Milk samples were tested in 2 conditions: the first condition without adding standard tetracycline and the second condition with adding standard tetracycline. For spiked milk samples, before being added with acetonitrile, the sample was added with standard tetracycline according to the detection limit results in the sensitivity test. Meanwhile, samples that were not spiked could be extracted directly.

A four‐mL milk sample suspected of containing tetracycline was added with 5 mL of acetonitrile and 200 μL of perchloric acid in a 15‐mL centrifuge tube and stirred for 1 min. Two grams of anhydrous Na_2_SO_4_ and 1 g of NaCl were added and mixed with a vortex mixer for 1 min and then centrifuged for 4 min at 10,000 rpm. An aliquot of 1 mL of the acetonitrile layer on top was transferred into a 2‐mL centrifuge bottle containing 0.5 mL of water and 0.5 mg of magnesium (II), and then vortexed for 1 min. About 1 M NaOH was added as much as 175 μL and vortexed for 30 s, and then centrifuged at 6500 rpm for 1 min. The precipitate formed was added to 10 μL of phosphoric acid and sonicated for 30 s. About 100 μL of 1 M NaOH was added again for the neutralization process. Deionized water was added until the final volume reached 0.2 mL [35].

### 2.4. Selection of Specific Reagents for Tetracycline Residue Detection

Specific chemical reagents used in this study were selected based on their ability to detect tetracycline by producing a permanent color change, selectivity, and ability to react to create a color change without heating. The color change follows the literature. Reagents, such as H_2_SO_4_, Mecke, Marquis, Zwicker, Mandelin, Froehde, Benedict, formaldehyde–sulfuric acid, thioxanthenes–sulfuric acid, and Lieberman, can be used to detect the presence of tetracycline residues. Zwicker, Froehde, Benedict, and formaldehyde–sulfuric acid reagents were not selected because they require a heating process to produce a color change. Mandelin and formaldehyde–sulfuric acid provide inconsistent color changes. In comparison, Liebeman’s manufacturing process requires cold temperatures. Therefore, concentrated H_2_SO_4_, Mecke, and Marquis reagents were selected because they met the desired criteria. Mecke, Marquis, and concentrated H_2_SO_4_ reagents provide color changes with tetracycline in purple, red, orange, and yellow, respectively [29].

### 2.5. Fabrication of Indicator Strips

**FIGURE 1:**
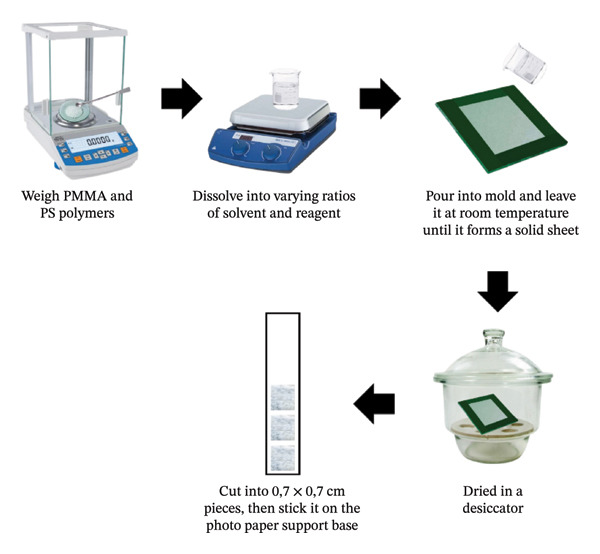
Schematic illustration for the fabrication of indicator strips.

The indicator strip was created using the reagent blending method, which involved dissolving PMMA polymer and a mixture of PS and PMMA in a ratio of 1:4 and 1:5 with a concentration of 5% in ethyl acetate using a magnetic stirrer at 350 rpm for 1 h and 15 min at room temperature. After dissolving, chemical reagents in varied ratios were added and agitated until homogeneous for 5 min. The solvent and chemical reagent ratios were 7:3, 8:2, and 9:1, respectively. The polymer liquid was placed over a glass whose edges had been constrained by foam tape with a mold size of 8 × 8 cm for a volume of 10 mL and then kept at room temperature for 1 h until the polymer liquid solidified [37–39]. The hardened polymer was then transferred to a Petri plate and dried in a desiccator for 24 h [40]. The indicator strip is divided into segments of 0.7 × 0.7 cm. The flow of making indicator strips is shown in Figure 1.

### 2.6. Characterization of Indicator Strips

Characterization aims to see changes in the surface structure of the strip after the polymer is mixed with the reagent caused by thermal stress, temperature, and humidity [41].

#### 2.6.1. Scanning Electron Microscope–Energy‐Dispersive X‐Ray Spectroscopy (SEM‐EDS)

The indicator strip was characterized using SEM‐EDS with magnifications of 2500x and 5000x. This technique aims to see the shape of the indicator strip’s pores and the mixing of the polymer with the reagent.

#### 2.6.2. IR Spectrophotometer

The solid indicator strip sample is placed on the ATR crystal. Then, the tool presses the sample to contact the ATR crystal directly. IR rays that pass through the crystal will experience total reflection, so some of the rays penetrate slightly into the sample, forming an evanescent wave. Some of the energy from the IR rays is absorbed or reflected into the crystal, which is then released from the crystal to the detector so that the absorbed and transmitted light can be detected, producing a spectrum that can be analyzed [42]. This shows the mixing between the polymer and the reagent, which is indicated by changes in the functional groups of the polymer before and after adding specific chemical reagents [43].

### 2.7. Performance Test of the Indicator Strip

The purpose of this test is to determine the indicator strip’s quality as a colorimetric analytical medium. There are four types of indicator strip performance tests for detecting tetracycline residues in animal products: selectivity, sensitivity, accuracy, and stability.

#### 2.7.1. Selectivity Test

To determine the selectivity of the specific chemical reagents contained in the indicator strip toward tetracycline, the indicator strip was tested with standard antibiotic groups other than tetracycline, the residues of which are also often found in animal products, including the penicillin group (ampicillin), aminoglycoside group (kanamycin sulfate and streptomycin sulfate), macrolides (tylosin), and fluoroquinolones.

#### 2.7.2. Sensitivity Test

The sensitivity test was performed to determine the lowest concentration that can be detected by an indicator strip. The indicator strip was dripped with tetracycline hydrochloride standard concentrations (1000, 500, 250, 100, 50, 40, 30, 20, 15, 10, and 5 ppm) of up to 20 μL each. The indicator strip was placed on white paper as a background, and the paper was positioned in a well‐lit room near a window, allowing sunlight to enter while avoiding shadows. Then, the indicator strips were placed against white background and documented using a smartphone camera (Samsung Galaxy A50 S, 48 Mpx) with a shooting distance of about 18 cm. It was saved in JPEG format and continued analysis using ImageJ [44]. The mean value indicating the color intensity creates a linear relationship curve between concentration and color intensity, which is measured to obtain a linearity equation and regression value [45].

Limit of detection (LOD) and limit of quantification (LOQ) are calculated to determine the detection limit of the indicator strip. The LOD and LOQ were calculated using a statistical approach based on the standard deviation (SD) of the tetracycline standard. The SD value was obtained using the following formula:

(1) Sy/x=∑y−ý2n−2,

where *S*(*y*/*x*) = the standard deviation of the response, *y* = real color intensity, ý = theoretical color intensity, and *n* = number of samples

The LOD and LOQ were determined based on the formulas outlined by ICH [46] as follows:

(2) LOD=3 x Sy/xS,

(3) LOQ=10 x Sy/xS,

where LOD = limit of detection, LOQ = limit of quantification, *S*(*y*/*x*) = the standard deviation of the response, and *S* = slope.

#### 2.7.3. Accuracy Test

Accuracy testing was conducted to measure the accuracy of the strip indicator in detecting tetracycline compared to other analysis methods that have been proven to be sensitive and valid, such as thin‐layer chromatography (TLC) and LC‐MS/MS [46]. Milk samples spiked with 15 ppm tetracycline were applied to the indicator strips containing the reagent. The color intensity was measured using ImageJ with a rectangular region of interest (ROI) of 265 × 265 pixels, and the concentration was then determined by inserting the intensity values into the linear regression equation obtained from the standard curve of each reagent‐based indicator strip. The calculated concentration was subsequently compared with the initial concentration added to the milk to obtain the percent recovery.

The silica gel plate was first saturated with methanol and dried in TLC testing. After that, the silica plate was saturated for 24 h with 0.1 M Na_2_EDTA solution with a pH of 8.0, which was adjusted by adding 40% NaOH and monitored with a pH meter. The plate was dried at 120°C for 1 h. The chamber was saturated in a mobile phase of chloroform:methanol:NH_4_OH 25% (60:35:5) for 15–30 min [47]. The GF254 silica gel plate was cut, and the upper and lower boundaries were marked with a pencil at a distance of 0.5 cm and 1 cm, respectively. Tetracycline sample and standard solutions were dripped onto the TLC plate using a capillary tube. The TLC plate was inserted into the chamber and left until the eluent touched the upper limit mark. After that, the TLC plate was lifted, dried, and observed under ultraviolet light at 254 nm and 366 nm to calculate the retention factor (Rf) value.

The test using LC‐MS/MS aims to identify tetracycline’s presence and measure the tetracycline levels in milk samples. The instrument used was LC‐MS/MS AB Sciex TQ 4500 with UPLC Waters Acquity I‐Class using an ACQ‐BEHC18 column. The gradient pump system used the mobile phase of 0.1% formic acid in aquabidest and methanol. The flow rate used was 0.25 mL/min. The samples of three goat and three cow milk samples were divided into two conditions: nonspike and spike. The extraction method is already available in the testing laboratory. The standard curve was made at a minimum of seven points. Five grams of the sample were mixed with the extractor buffer in a 50‐mL Falcon tube. Then, it was vortexed and shaken with a mechanical shaker. Furthermore, the solution was centrifuged, and the supernatant was transferred into another Falcon tube. The stage of adding the extractor buffer and centrifugation was repeated, and then, the supernatant was transferred to the same tube. The extractor buffer solution was added back to the sample and vortexed. Then, it was shaken with a mechanical shaker and centrifuged. The supernatant was transferred into the same Falcon tube and centrifuged again. The supernatant was filtered with microfiber and rinsed with an extractor solution. Clean‐up was done using solid‐phase extraction (SPE), which was collected in a measuring flask. Then, aquabidest was added to the boundary mark and homogenized. The sample was filtered with a syringe filter and injected into LC‐MS/MS. The absorbance of the tetracycline sample is entered into the linear equation of the tetracycline standard solution to obtain the tetracycline concentration of the sample.

#### 2.7.4. Stability Test

The indicator strip is stored in a dark glass bottle in a desiccator, closed, dry, and protected from light [40]. This test is carried out by dripping a standard tetracycline solution daily. Stability was then observed until the indicator strip did not produce a color change or a positive result.

## 3. Results and Discussion

### 3.1. Sample Collection and Preparation

The selection of the closest place was to maintain the stability of the milk samples during shipping at room temperature because the pH of the milk will decrease more at a temperature of 26°C for 24 h compared to milk stored in the refrigerator [48]. pH is one of the factors that can affect the stability of tetracycline, so a unique extraction process is required to adjust the pH so that tetracycline can be perfectly extracted. All samples have milky white organoleptic properties, with two samples from Bogor and four from Bandung taken directly from the farm. The samples were stored in a freezer at a temperature of less than −17°C to keep the milk fresh and prevent bacterial contamination that can damage the beneficial substances contained in the milk [49].

The samples were separated into two groups: The first was spiked with 15 ppm standard tetracycline, and the second was not. The concentration of the standard spike was determined by the LOQ obtained from ImageJ analysis on the indicator strip. All samples were removed from the freezer and thawed first by immersing them in a water bath at room temperature. The samples were first added with acetonitrile to dissolve the tetracycline [50]. Perchloric acid was added to release the tetracycline bond and precipitated the protein [51]. Sodium sulfate (Na_2_SO_4_) was used to draw excess water from the milk sample so as not to interfere with the analysis process [52]. The addition of NaCl functions to precipitate compounds that are more soluble in water [53]. A vortex assists this process to increase contact and interaction between the sample and the additive. Separating tetracycline from the sample is optimized by centrifugation to form two phases consisting of sediment and supernatant [54].

The upper layer, in the form of acetonitrile liquid, is transferred to another tube and mixed with magnesium sulfate solution, which functions in the complex formation process. The solution is adjusted in primary conditions (pH 11) by adding NaOH to change the form of tetracycline into an anion that can chelate magnesium metal [35]. In this condition, tetracycline will form a white Mg(OH)_2_ precipitate optimized by centrifugation [55]. Weak acidic conditions are needed with the addition of phosphoric acid to the precipitate, which functions to change the structure of tetracycline into a cation so that it can release tetracycline from magnesium and dissolve again [56]. Tetracycline can be degraded at very low (pH < 2) or very high (pH 11) pH conditions [57, 58]. Therefore, strong acids, such as perchloric acid, are not recommended for reuse under these conditions. NaOH was added to neutralize tetracycline to pH 7 to be analyzed in a zwitterion state [59].

### 3.2. Selection of Specific Reagents for Tetracycline Residue Detection

Mecke, Marquis, and H_2_SO4 reagents give positive color changes to tetracycline, as shown in Table 1. Mecke’s reagent is used to identify alkaloid groups and psychoactive drugs, such as 3,4‐methylenedioxy‐N‐methylamphetamine (MDMA). Meanwhile, the Marquis reagent can identify alkaloid, opiate, and amphetamine groups [29]. Concentrated H_2_SO_4_ reagent is a strong oxidizer that detects organic and inorganic compounds, mainly phenols and alcohols [60]. Tetracycline can be identified with Mecke and Marquis reagents even though it is not included in the alkaloid, opiate, and amphetamine groups because it has a fused ring and complex side groups that are rarely found in other compounds. Tetracycline has four connected carbon rings (rings A, B, C, and D) called fused rings [61]. On the ring, there are various complex functional groups, such as hydroxyl (OH), amine (NH_2_), and ketone (C=O) groups. Hydroxy and ketone groups can cause keto‐enol tautomerization, which is very reactive to strong oxidants [62, 63]. Amine groups can affect interactions with hydrogen molecules in strong acids through electronic effects [64].

**TABLE 1:** Results of color changes of specific chemical reagents with 1000 ppm tetracycline standard.

Blank reagent	References	Documentation	Result
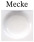	Purple red	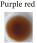	+

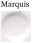	Orange	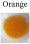	+

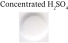	Yellow	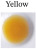	+

*Note:* Information: (−): tetracycline not detected; (+): tetracycline detected.

### 3.3. Fabrication of Indicator Strips

The polymer manufacturing method used in making indicator strips is the reagent blending method. This method is carried out by mixing polymers, solvents, and specific chemical reagents. This study tested several formulations using PMMA and PS polymers to obtain an indicator strip composition that can provide appropriate, fast, sensitive, and selective color changes. In the experiment, there was a combination formulation of PMMA and PS polymers, so it was necessary to select a solvent to produce a homogeneous solution.

The Hildebrand solubility parameter can be used to determine the appropriate solvent in dissolving polymers based on cohesion energy. Cohesion is the attractive force between similar molecules in a substance. In contrast, cohesion energy is the amount of energy needed to separate one molecule from another molecule of a substance so that the molecules separate, causing the surface tension to decrease, the boiling point and melting point to decline, the vapor pressure to increase, and the solubility to increase [65, 66]. Similar Hildebrand values have similar polarity properties and similar types of intermolecular interactions so they will increase solubility [67]. PMMA and PS polymers are soluble in chloroform, ethyl acetate, acetone, benzene, butyl acetate, toluene, methyl ethyl ketone, tetrahydrofuran, trichloroethylene, and xylene [68]. Table S1 in the supporting information shows the Hildebrand values of polymers and solvents. PMMA has a close Hildebrand value with ethyl acetate, while PS is closer to trichloroethylene. The difference in the Hildebrand solubility parameter values of PS and ethyl acetate is insignificant, only 0.2 [cal^1/2^ cm^−3/2^], so PS can still dissolve in these solvents [69].

One factor that affects the indicator strip’s structure is the polymer concentration [70]. The polymer concentration is closely related to the size of the pores formed. Research conducted by Satiti [71] stated that a polymer concentration of 2.5% produces a fragile indicator strip and large pore size, making it difficult to use. A polymer concentration of 7.5% forms an indicator strip with too small pores, causing the specific chemical reagents retained in the indicator strip to be small. Meanwhile, a polymer concentration of 5% produces a strong indicator strip, and the pores are not too small. This study conducted experiments with PMMA, PS, and a combination of PS: PMMA polymers. The PS: PMMA composition ratio of 1:2 and 1:3 formed a less homogeneous, thin, and fragile indicator strip. Meanwhile, the composition of 1:4 has fairly good pores and is stronger than the previous comparison [38]. The PS: PMMA 1:5 and 1:6 composition also produced a more robust indicator strip than without the combination [34]. Therefore, the composition of PS: PMMA 1:4 and 1:5 was used in this study. The amount of PS used should not be too much because it is hydrophobic or does not absorb water so that it can inhibit the interaction between specific chemical reagents and active substances [72].

PS polymer shows a longer dissolution time compared to PMMA. This is because the Hildebrand solubility parameter value is more significant than ethyl acetate. Meanwhile, the difference in the Hildebrand solubility parameter of PMMA is smaller than that of ethyl acetate. The time required for PMMA to dissolve in ethyl acetate is 1 h, while PS takes 1 h to 15 min to dissolve in ethyl acetate. No heat is used to dissolve the polymer because it is feared that it can damage the specific chemical reagents that will be mixed into the polymer solution. After the polymer dissolves, the chemical reagents are mixed into the solution with a ratio of 7:3, 8:2, and 9:1 for 5 min at 350 rpm and room temperature. When the reagent is mixed into the polymer solution, an exothermic reaction occurs, which is indicated by the emergence of heat. Printing the polymer solution needs to be done with the appropriate mold size so that the thickness of the indicator strip is uniform. After experimenting, it was found that the mold size of 8 × 8 cm was suitable for a solution volume of 10 mL and a polymer concentration of 5%, producing an indicator strip with a thickness of 0.3 mm.

PMMA polymer is hygroscopic, and it must be dried in a room temperature desiccator that can absorb environmental moisture [40, 73]. The indicator strip’s 24‐h storage duration in a desiccator results in a dry sheet surface while still providing adequate and quick color changes. Dropping 20 μL of a 1000 ppm tetracycline solution on the surface of the 0.7 cm × 0.7 cm indicator strip results in a quick color shift, as indicated in Table 2.

**TABLE 2:** Results of indicator strip design from PMMA and PS:PMMA polymer with the reagent blending method.

Polymer	Reagent	Ratio of ethyl acetate and reagent	Indicator strip shape	Result	Analysis time
PMMA 5%	Mecke	7:3	Not formed	−	−
		8:2	Not formed	−	−
		9:1	Sheet	+	10 s
	Marquis	7:3	Not formed	−	−
		8:2	Not formed	−	−
		9:1	Sheet	+	10 s
	Concentrated H_2_SO_4_	7:3	Not formed	−	−
		8:2	Not formed	−	−
		9:1	Sheet	+	10 s

PS: PMMA (1:4) 5%	Mecke	7:3	Not formed	−	−
		8:2	Not formed	−	−
		9:1	Sheet	+	39 min
	Marquis	7:3	Not formed	−	−
		8:2	Sheet	+	39 min
		9:1	Sheet	+	55 min
	Concentrated H_2_SO_4_	7:3	Sheet	+	10 min
		8:2	Sheet	+	7 min
		9:1	Sheet	+	39 min

PS: PMMA (1:5) 5%	Mecke	7:3	Not formed	−	−
		8:2	Sheet	+	55 min
		9:1	Sheet	+	55 min
	Marquis	7:3	Not formed	−	−
		8:2	Sheet	+	55 min
		9:1	Sheet	+	55 min
	Concentrated H_2_SO_4_	7:3	Not formed	−	−
		8:2	Sheet	+	55 min
		9:1	Sheet	+	39 min

*Note:* Information: (+): tetracycline detected; (−): no data.

The PMMA (9:1), PS: PMMA 1:4 (9:1), and PS: PMMA 1:5 (9:1) indicator strips for specific chemical reagents Mecke, Marquis, and concentrated H_2_SO_4_ were chosen because they formed better sheets, suitable color changes, and the fastest analysis time compared to other formulas. The color change on the indicator strip appears dimmer than on the dropper plate because the reagent volume is small and evenly distributed on the indicator strip, so the volume is even smaller. PMMA is not resistant enough to strong acids in large quantities compared to PS. However, PMMA is still resistant to strong acids in small amounts. PMMA mixed with dilute sulfuric acid concentrations causes the polymer to clump, and its thickness is uneven. The analysis time for color changes on PS:PMMA gives longer results than for PMMA, which only needs 10 s for color changes because the hydrophobic PS layer on the surface of the indicator strip makes contact between the analyte and the reagent, and visualization of color changes is blocked [72]. Based on these findings, PMMA was chosen for further investigation.

### 3.4. Characterization of Indicator Strips

#### 3.4.1. SEM‐EDS

**FIGURE 2:**
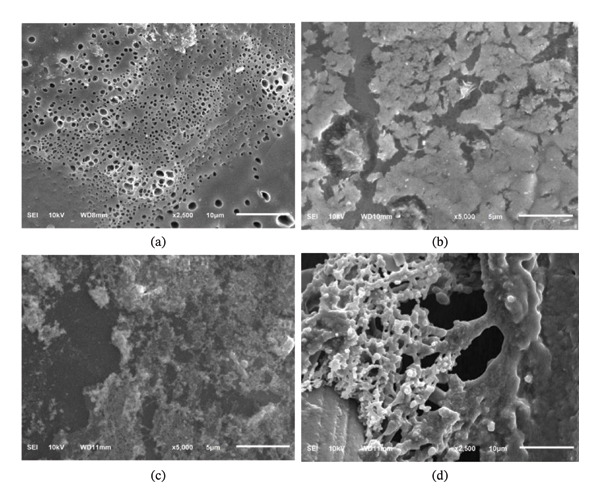
PMMA microstructures: (a) PMMA (2500x); (b) PMMA‐concentrated H_2_SO_4_ (5000x); (c) PMMA‐Mecke (5000x); (d) PMMA‐Marquis (2500x).

The morphology of the indicator strip was observed using SEM at 2500x and 5000x magnification. EDS aims to observe the mixing of polymers with reagents, proven by the instrument’s detection of the elements that make up the reagents. The results are shown in Figure 2.

PMMA has a more homogeneous structure compared to PMMA mixed with the reagents Mecke, concentrated H_2_SO_4_, and Marquis. This is due to the interactions between the reagents and the PMMA polymer. The reagents contain concentrated sulfuric acid, a strong oxidizer, which induces chemical changes in the PMMA structure accompanied by the release of heat. In addition, the reagents can penetrate the small gaps between the PMMA chains, pushing the polymer chains apart. The polar groups present in PMMA allow the polymer to absorb the polar molecules contained in the reagents. As a result, PMMA swells slightly but does not break down or dissolve. This phenomenon makes PMMA thicker, more porous, and capable of retaining the reagents. The irregular cavities formed lead to uneven color changes.

EDS is a spectroscopic technique used to analyze the chemical composition of samples observed with SEM [74]. This technique works by measuring the energy of X‐rays produced by the sample when exposed to electrons from SEM [75]. X‐ray energy will produce output in the form of a spectrum that provides information about the presence of chemical element concentrations [76]. The presence and concentration of elements contained in the indicator strip are shown in Table S2 in the supporting information.

The color change on the indicator strip is based on the reaction between the chemical elements of the specific reagent and the tetracycline functional group. Therefore, the mixed reagent must be ensured to have entered the pores of the PMMA. From the results of the EDS analysis, the specific chemical reagent entered the pores of the indicator strip, which is indicated by the presence of chemical elements detected on the device [34]. Mecke’s reagent contains concentrated sulfuric acid and selenium acid, as evidenced by sulfur and selenium elements. Marquis reagent contains concentrated sulfuric acid and formaldehyde (CH_2_O), as indicated by sulfur, carbon, and oxygen elements. Sulfur elements on the indicator strip indicate concentrated sulfuric acid reagent.

#### 3.4.2. IR Spectrophotometer

Characterization with IR spectrophotometry aims to analyze the presence of polymer functional groups and reagents mixed with the polymer. It is carried out at a wavelength of 4000‐450 cm^−1^. The analysis results of functional groups before and after mixing with specific reagents are shown in Figure S1 in the supporting information.

Based on the results of the analysis, the indicator strip mixed with specific reagents (Mecke, Marquis, and concentrated H_2_SO_4_ reagents) caused several changes in intensity and peak shifts [38]. PMMA polymer mixed with a reagent containing concentrated sulfuric acid causes the peak intensity to weaken at wave numbers 2900 to 400 cm^−1^. This is because there is an exothermic reaction between PMMA, solvent, and reagent, which causes several bonds of PMMA functional groups to break, which can reduce the adsorption of IR rays.

New peaks appear after the reagent is mixed with PMMA. At wave numbers around 1170, 1060, 880, and 610 cm^−1^, each shows the peaks of O=S=O asymmetric, O=S=O symmetric, ${\text{HSO}}_{4}^{-}$, and S‐O stretching [77–79]. The detection of these bonds indicates that the reagent has entered the indicator strip. Hydroxyl functional groups were detected in the PMMA‐based indicator strip because PMMA can be hygroscopic and absorb environmental moisture [80].

### 3.5. Reaction Mechanism of Reagents—PMMA for Tetracycline Detection

The structure of PMMA consists of linear, amorphous polymer chains formed from methyl methacrylate monomers. Its polarity arises from the ester groups (–COOCH_3_) present in each monomer unit, which render PMMA polar [81]. Mecke, Marquis, and concentrated H_2_SO_4_ contain sulfonic acid groups with hydroxyl (–OH) groups composed of highly electronegative oxygen atoms and highly electropositive hydrogen atoms [82]. Mecke also contains selenious acid, which has –OH groups and highly electronegative Se–O bonds. This electron imbalance makes both sulfuric acid and selenious acid highly polar. Marquis also contains formaldehyde, which has highly electronegative oxygen atoms. This creates a large dipole in the C–O bond.

The reagents penetrate the small gaps between the PMMA polymer chains. The polar molecules push the polymer chains apart. The polar groups present in PMMA allow the polymer to absorb other polar molecules contained in the reagents, causing slight swelling without damaging or dissolving the polymer [83]. This phenomenon makes PMMA thicker, more porous, and capable of retaining the reagents. Tetracycline has polar functional groups, such as –OH, –NH, and ketone, which allow it to be absorbed and diffuse into the pores of PMMA. Tetracycline then interacts with the reagents retained within the PMMA matrix.

The reaction between tetracycline and Mecke’s reagent changes to purple–red color. The mechanism of this reaction is not yet clearly known because there are several differences in the functional groups bound to tetracycline with other compounds, such as opiates, which can also be analyzed with Mecke’s reagent. In general, the reaction mechanism between Mecke and opiates has been known, where concentrated sulfuric acid breaks the bonds in the methoxy functional group to react with selenium to stabilize the structure of the compound [84].

The reaction between tetracycline and Marquis reagent produces an orange color change. The nucleophilic aromatic ring of tetracycline attacks the aldehyde carbon of formaldehyde to form a primary benzyl carbocation. This structure is unstable, so it is attacked again by a second nucleophile to produce a new bond that causes the positive charge on the carbon atom to disappear. Concentrated sulfuric acid oxidizes the structure, resulting in the addition of oxygen. Furthermore, the structure undergoes dehydration to form a stable secondary benzyl carbocation [85].

The reaction between tetracycline and concentrated H_2_SO_4_ reagent produces a yellow color change. Tetracycline in acidic conditions undergoes dehydration, namely the loss of the hydroxyl group at C6. The oxygen atom from the hydroxyl group C6 attacks the H+ group from concentrated sulfuric acid to form a protonated hydroxyl group. A tertiary carbocation is formed when the protonated hydroxyl group breaks from the bond. The hydrogen atom on the carbon adjacent to the carbocation gives its electrons to the carbocation so that the single bond changes to a double bond. This conformation is stabilized by transferring oxygen electrons to the hydroxyl group next to the ketone. The hydroxyl group changes into a ketone and then changes into OH, and the double bond moves [61].

### 3.6. Performance Test of the Indicator Strip

#### 3.6.1. Selectivity Test

The selectivity test aims to determine that the chemical reagents Mecke, Marquis, and concentrated H_2_SO_4_ only provide specific color changes to detect tetracycline residues compared to other drug residues often found in animal products. Selectivity testing was carried out on tetracycline, ampicillin, kanamycin, streptomycin sulfate, enrofloxacin, and standard tylosin. Several antibiotics, such as tetracycline, enrofloxacin, and tylosin, can chelate metals to be extracted during the sample preparation process [86].

Based on the results of the selectivity test in Table S3 in the supporting information, it was found that the Mecke, Marquis, and H_2_SO_4_ reagents gave a specific color change for tetracycline only. This is evidenced by the reaction between the reagents with ampicillin, kanamycin, streptomycin sulfate, enrofloxacin, and tylosin, which did not give the exact color change as tetracycline. While this method demonstrates high selectivity against different antibiotic classes, further studies are required to evaluate the cross‐reactivity among various tetracycline derivatives to fully establish its specific recognition profile.

#### 3.6.2. Sensitivity Test

The first step that must be taken in analyzing the indicator strip using ImageJ is to adjust the hue, saturation, and value (HSV). Hue functions to change the color spectrum to be analyzed. Saturation indicates the amount of white mixed with color. The value in HSV indicates the intensity or brightness of the color [87]. HSV adjustment is carried out until the blank indicator strip composed of PMMA and specific reagents does not show an absorption value when measured. The hue, saturation, and brightness values ​​obtained are 44, 63, and 0, respectively. The next step is to invert the color of the indicator strip after adjusting the HSV, which aims to see the contrast between the adjusted and unadjusted colors so that color changes can be seen clearly. From the picture, it can be seen that there is a difference in the color intensity produced in the concentration variations. The highest concentration shows a very bright color, and its intensity decreases as the concentration of standard tetracycline dropped on the indicator strip decreases.

**FIGURE 3:**
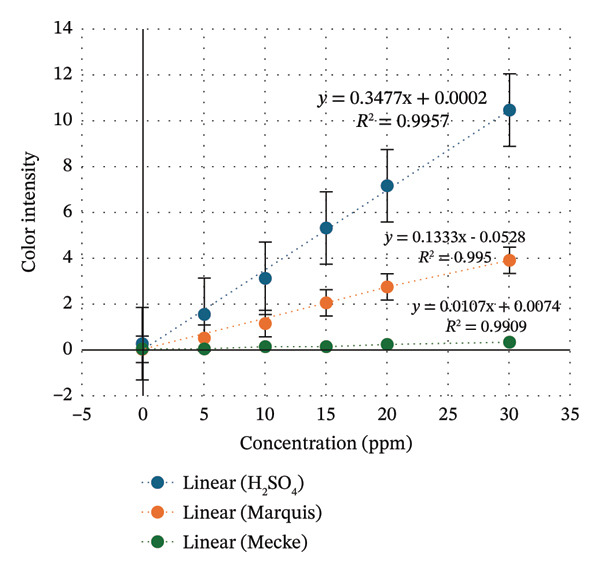
Sensitivity curve of indicator strip containing H_2_SO_4_, Mecke, and Marquis reagents.

The linearity relationship curve plots the color intensity value against the standard tetracycline concentration. Furthermore, calculations are carried out to determine the LOD and LOQ. The higher the concentration, the greater the color intensity produced, so the calibration curve and line equation are obtained, as shown in Figure 3.

LOD is the lowest concentration of analyte that can produce a visually distinguishable color change. In contrast, LOQ measures the lowest concentration of analyte that can provide accurate and precise numerical values. The results show the LOD of Mecke’s, Marquis, and H_2_SO_4_ were 3.49, 2.57, and 2.38 ppm, respectively. In addition, the LOQ was 11.64, 8.57, and 7.95, respectively. The results show that the LOD obtained for this indicator strip is 3.49 ppm, while the LOQ is 11.64 ppm. This result was chosen based on the highest result of determination because in the design of the indicator strip, samples at specific concentrations are dripped onto the three indicator strips simultaneously.

#### 3.6.3. Accuracy Test

Accuracy testing was conducted to measure the accuracy of the indicator strip in detecting tetracycline compared to more sensitive, selective, and precise instruments, such as TLC and LC‐MS/MS. The extracted milk samples were subjected to initial TLC screening to detect tetracycline’s presence qualitatively. Silica gel GF254 was used as the stationary phase, and a mixture of chloroform: methanol: 25% NH_4_OH (60:35:5) was used as the mobile phase. This combination of mobile phases was chosen because it can separate tetracycline from other matrices without tailing so that the Rf value can be appropriately determined [47]. Analysis of tetracycline using TLC can produce tailing because tetracycline can bind to silanol groups at pH above 7.7 [88]. Therefore, silica gel GF254 must first be soaked in EDTA with a pH of 8 to avoid tetracycline interaction with silanol [89]. Soaking silica gel GF254 for 24 h can provide good analysis results [90]. EDTA can bind to cations at pH 8 to 10 [91].

**FIGURE 4:**
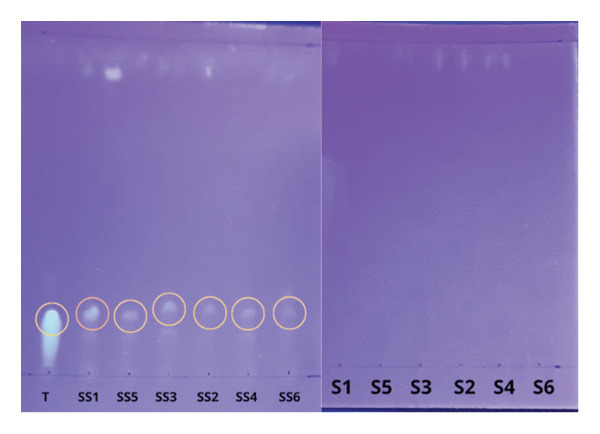
TLC results of milk samples compared with tetracycline standards under 366 nm UV lamp (T: standard tetracycline; S1, S2, S3, S4, S5, and S6: nonspike sample; SS1, SS2, SS3, SS4, SS5, SS6: spike sample).

In determining the presence of tetracycline in the sample, the tetracycline standard solution used as a comparison is also spotted so that the Rf value can be compared with the sample, as shown in Figure 4 and Table S4 in the supporting information. The tetracycline structure changes to an anion form and can chelate metals at pH > 9.7 [59]. The pH of the solution should not be too acidic so as not to damage the silica gel GF254. Before screening, the pH of the tetracycline standard is adjusted to a neutral pH (pH 7), the same as the pH of the sample, so that the resulting Rf is the same.

All milk samples screened using TLC did not show any spots indicating the presence of tetracycline when analyzed with a 366 nm UV lamp. However, samples spiked with tetracycline standards showed the presence of tetracycline spots. In TLC, a sample is declared positive if the Rf difference between the standard and the sample is ≤ 0.05 [92]. The spot has an Rf value of ≤ 0.05, different from the tetracycline standard, so the extraction process is proven to separate tetracycline from the sample. Tetracycline, set at pH 7, still produces tailing, so the pH must be lowered to around pH 5 to 6. The pH should not be too acidic because it can damage silica. Samples that LC‐MS/MS had previously confirmed showed the presence of tetracycline in tiny concentrations. However, samples analyzed by TLC did not show the presence of tetracycline. This is because the final result of the sample extraction is not very concentrated. Hence, the concentration of tetracycline in the sample is deficient, and other matrices from the solution disrupt the analysis process. LC‐MS/MS instrument is used to analyze the presence and levels of tetracycline residues in milk samples. This instrument is chosen because it is widely used and can detect tetracycline residues at low concentrations in samples with complex matrices.

Table S5 in the supporting information shows that the levels of tetracycline residue obtained are within the tolerance limits permitted by the EU, FAO, and SNI. The MRL of tetracycline permitted by the EU and FAO is a maximum of 0.1 ppm and, according to SNI, is 0.05 ppm. The extracted milk sample was then dripped onto the indicator strip to see the effectiveness of the indicator strip in detecting tetracycline in the sample. After that, the color change was observed using ImageJ, and the color intensity was measured and entered into the standard. The color intensity produced by the interaction of tetracycline from the sample with the reagent at the spiked tetracycline concentration must provide the same intensity as the tetracycline standard that has been dripped onto the indicator strip. The sample analysis results with ImageJ are shown in Table 3.

**TABLE 3:** Results of sample analysis on indicator strips containing Mecke’s, Marquis, and H_2_SO_4_ reagents using ImageJ.

Sample code	Color intensity			Spike concentration (ppm)	Detected concentration (ppm)			Recovery (%)		
	Mecke’s	Marquis	H_2_SO_4_		Mecke’s	Marquis	H_2_SO_4_	Mecke’s	Marquis	H_2_SO_4_
SS1	0.17	1.85	4.99	15	14.92	14.24	14.35	99.47	94.93	95.67
SS2	0.16	1.85	5.00	15	13.89	14.23	14.38	92.60	94.87	95.87
SS3	0.14	1.94	4.93	15	12.49	14.95	14.19	83.27	99.66	94.60
SS4	0.17	1.89	5.04	15	14.92	14.53	14.50	99.44	96.87	96.67
SS5	0.15	1.74	5.13	15	13.70	13.42	14.76	91.33	89.47	98.40
SS6	0.15	1.87	5.07	15	13.05	14.87	14.59	87.00	99.16	97.27

The sample analysis on the indicator strip showed a change in color intensity close to the standard tetracycline 15 ppm that had been analyzed previously. The color intensity produced in the sample was entered into the standard curve formula of each indicator strip. The calculation found that the concentration of tetracycline measured by ImageJ gave good recovery results, namely 83.27%–99.66%. The wide range of the recovery can be caused by uneven color distribution in paper surface due to many cavities identified by SEM.

Based on the result, a preliminary comparison revealed a considerable difference between the two methods’ measured concentrations. Results from the LC‐MS/MS analysis as shown in Table S5 ranged from 7.38 to 10.20 ppm, which is a significant decrease from the original 15 ppm spike concentration. However, the indicator strip showed excellent internal accuracy, with a recovery range of 83.24%–99.66% in relation to the spiking concentration. This is most probably caused by difference in the sample preparation and extraction protocols utilized by the external LC‐MS/MS service. It is hypothesized that the external extraction procedure resulted in an incomplete recovery of the tetracycline analyte from the milk matrix. Therefore, the validation of the proposed method focuses on the high recovery and accuracy demonstrated against the known spiked concentration, which confirms the indicator strip’s suitability for rapid quantitative screening.

However, the recovery tests in this study were conducted at a spiked concentration of 15 ppm, which aligns with the method’s LOQ of 11.64 ppm. Due to this technical detection threshold, performing recovery trials at the regulatory MRL level (0.05–0.1 ppm) was not feasible for the current method. Consequently, this indicator strip is designed as a rapid screening tool specifically for detecting high‐level tetracycline contamination. Potential application scenarios include on‐field preliminary screening for gross contamination—such as cases involving extreme veterinary drug overdoses—and rapid verification in educational or industrial settings where high‐concentration detection is required. Additionally, it serves as an efficient tool for scenarios requiring the monitoring of intentionally spiked samples for specific experimental purposes.

#### 3.6.4. Stability Test

The stability test measures the indicator strip’s ability to maintain the reagent during storage. The reagent indicator strip is stored in a tightly closed brown glass bottle. This is to prevent the evaporation of sulfuric acid and degradation of the reagent by light. PMMA is hygroscopic and can absorb water from the environment, so it must be stored in a desiccator at room temperature. The water absorption by PMMA is feared to reduce the effectiveness and sensitivity of the reagent in detecting tetracycline. The indicator strip must be kept away from sources of fire or places that are too hot to prevent explosions or fires originating from sulfuric acid. The indicator strip was observed for color changes until the 60th day, and the test results showed that it could still show color changes until that day. This provides information that the indicator strip is still stable until that day in inappropriate storage.

To determine the advantages and disadvantages of the colorimetric technique, some data are offered as a comparison in Table 4. The findings indicate that polymer‐based colorimetric techniques can be used as an alternative for detecting an active chemical. This technique has several advantages, including the lack of expensive specialized equipment, quick detection, portability, ease of use, onsite application, and the requirement for a small sample amount. Although this study was less sensitive to detecting very low amounts, it did offer a quick color change with reasonably good recovery. This work still needs further refinement, primarily in selectivity for additional tetracycline derivatives as well as sensitivity.

**TABLE 4:** Comparison of research results with biosensor methods.

Sample type	Analyte	Method	Response time	LOD/LOQ	RSD/recovery (%)	References
Milk	Tetracycline, oxytetracycline, chlortetracycline, and doxytetracycline	Lateral‐flow immunochromatographic (LFIC) using upconverting nanoparticles (UCNPs)	60 min	—	RSD = ≤ 9.95 Recovery = 93.95–111.9	[18]

Honey, milk, meat, and egg	Tetracycline	Lateral‐flow immunochromatographic (LFIC) using EuNPs‐FIA	15 min	LOD = 0.12–2.39 ng/mL	Recovery = 100.83–106.1	[93]

Milk	Oxytetracycline, tetracycline, and chlortetracycline	Lateral‐flow immunochromatographic (LFIC) using AuNPs	5 min	LOD = 0.045–0.18 μg/mL LOQ = 0.075–0.2 μg/mL	RSD = 0.21–2.07 Recovery = 84.75–105.25	[94]

Milk, serum, and urine	Oxytetracycline	Lateral‐flow immunochromatographic (LFIC) using AuNPs	6–15 min	LOD = 15 μg/L	—	[95]

Milk	Tetracycline	Multiple lateral‐flow immunochromatographic (LFIC) using AuNPs	—	LOD = 0.082 ng/mL	Recovery = 107.1–166.6	[96]

Milk	Tetracycline	Polymer‐based	10 s	LOD = 3.49 μg/mL LOQ = 11.64 μg/mL	RSD = 0.0012–0.0058 Recovery = 83.24–99.66	This work

## 4. Conclusion

The composition of PMMA polymer 5% with a solvent and reagent ratio of 9:1, including PMMA‐Mecke (9:1), PMMA‐Marquis (9:1), and PMMA‐H_2_SO_4_ (9:1), is the best as a base material for indicator strips to detect tetracycline residues in milk. PMMA‐based indicator strips that have been mixed with reagents produce an IR peak intensity that is weaker than before being mixed due to the exothermic reaction. An IR spectrophotometer detects the bond of the reagent molecules. EDS also detects the reagent elements in the indicator strip, indicating that the reagent has entered the indicator strip. SEM testing shows that the pores of the PMMA‐based indicator strip mixed with reagents become inhomogeneous. The indicator strip for detecting tetracycline residues has a LOQ of 11.64 ppm and LOD of 3.49 ppm. The indicator strip is stable for use for 60 days. In addition, this indicator strip has good accuracy in detecting tetracycline in milk samples with concentrations above the LOQ using ImageJ of 83.24%–99.66%. The indicator strip might potentially be used as a screening tool for high‐level tetracycline contamination, yet not for regulatory compliance. Future development will focus on enhancing the sensitivity through preconcentration steps to approach regulatory MRL levels.

## Author Contributions

Rimadani Pratiwi: conceptualization, methodology, writing–original draft, writing–review and editing, visualization, supervision, and funding acquisition. Putri Nur Azizah: data curation, investigation, writing–original draft, writing–review and editing, and visualization. Aliya Nur Hasanah: conceptualization, methodology, and writing–review and editing.

## Funding

This study was funded by the National Report and Innovation Agency (BRIN) and Educational Fund Management Institution (LPDP) through Riset dan Inovasi untuk Indonesia Maju (RIIM) grants 2023 number 5994/UN6.3.1/PT.00/2023.

## Disclosure

The presentation of the manuscript is a preprint in the following link: https://www.researchgate.net/publication/387904071_Rapid_Detection_of_Tetracycline_Residues_in_Milk_Using_Colorimetric_Sensor_Based_on_Polymethylmethacrylate_Pmma_and_Polystyrene_Ps_Polymers.

## Conflicts of Interest

The authors declare no conflicts of interest.

## Supporting Information

Additional supporting information can be found online in the Supporting Information section.

## Supporting information


**Supporting Information** Table S1. Hildebrand solubility parameters of polymers and solvents (Burke, 2015). Table S2. Mass percentage of chemical elements in indicator strip. Figure S1. Comparison of the infrared spectrum of (A) PMMA and the spectrum of PMMA mixed with Mecke′s Reagent, (B) PMMA and the spectrum of PMMA mixed with Marquis reagent, and (C) PMMA with the spectrum of PMMA mixed with concentrated H2SO4 reagent. Table S3. Selectivity test results of indicator strips against tetracycline. Table S4. Comparison of Rf values of TLC screening results of tetracycline standards. Table S5. Results of measurement of tetracycline residue levels in spiked and unspiked.

## Data Availability

The data supporting the findings of this study are included in this published article and its supporting information files.
