# Supplementary material for: Rapid Detection of Tetracycline Residues in Milk Using Colorimetric Sensor Based on Polymethylmethacrylate (PMMA) and Polystyrene (PS) Polymers
Source: J Anal Methods Chem. 2026 May 20;2026:5412774. doi: 10.1155/jamc/5412774 (PMC13189459; doi:10.1155/jamc/5412774)
Supplement: Supplementary file 1 — Supporting Information Table S1. Hildebrand solubility parameters of polymers and solvents (Burke, 2015). Table S2. Mass percentage of chemical elements in indicator strip. Figure S1. Comparison of the infrared spectrum of (A) PMMA and the spectrum of PMMA mixed with Mecke′s Reagent, (B) PMMA and the spectrum of PMMA mixed with Marquis reagent, and (C) PMMA with the spectrum of PMMA mixed with concentrated H2SO4 reagent. Table S3. Selectivity test results of indicator strips against tetracycline. Table S4. Comparison of Rf values of TLC screening results of tetracycline standards. Table S5. Results of measurement of tetracycline residue levels in spiked and unspiked. [file JAMC-2026-5412774-s001.docx]

**SUPPLEMENTARY INFORMATION**

**Table S1.** Hildebrand Solubility Parameters of Polymers and Solvents (Burke, 2015)

| **Polymer/Solvent** | **Hildebrand Values (cal^1/2^ cm^−3/2^)** |
| --- | --- |
| Polymethyl methacrylate (PMMA) | 9.1 |
| Polystyrene (PS) | 9.3 |
| Ethyl acetate | 9.1 |
| Chloroform | 9.21 |
| Acetone | 9.77 |
| Benzene | 9.15 |
| Butyl acetate | 8.5 |
| Toluene | 8.91 |
| Methyl ethyl ketone | 9.27 |
| Tetrahydrofuran | 9.52 |
| Trichloroethylene | 9.28 |
| Xylene | 8.85 |

**Table S2.** Mass Percentage of Chemical Elements in Indicator Strip

| **Indicator strip Types** | **Element** | **%Mass** |
| --- | --- | --- |
| PMMA 5% | Carbon | 89,94 |
|  | Oxygen | 10,06 |
| PMMA-Mecke 9:1 (5%) | Carbon | 56,07 |
|  | Oxygen | 7,89 |
|  | Sulfur | 32,98 |
|  | Selenium | 3,05 |
| PMMA-Marquis 9:1 (5%) | Carbon | 58,37 |
|  | Oxygen | 10,11 |
|  | Sulfur | 31,53 |
| PMMA-Concentrated H_2_SO_4_ 9:1 (5%) | Carbon | 52,18 |
|  | Oxygen | 10,72 |
|  | Sulfur | 37,10 |


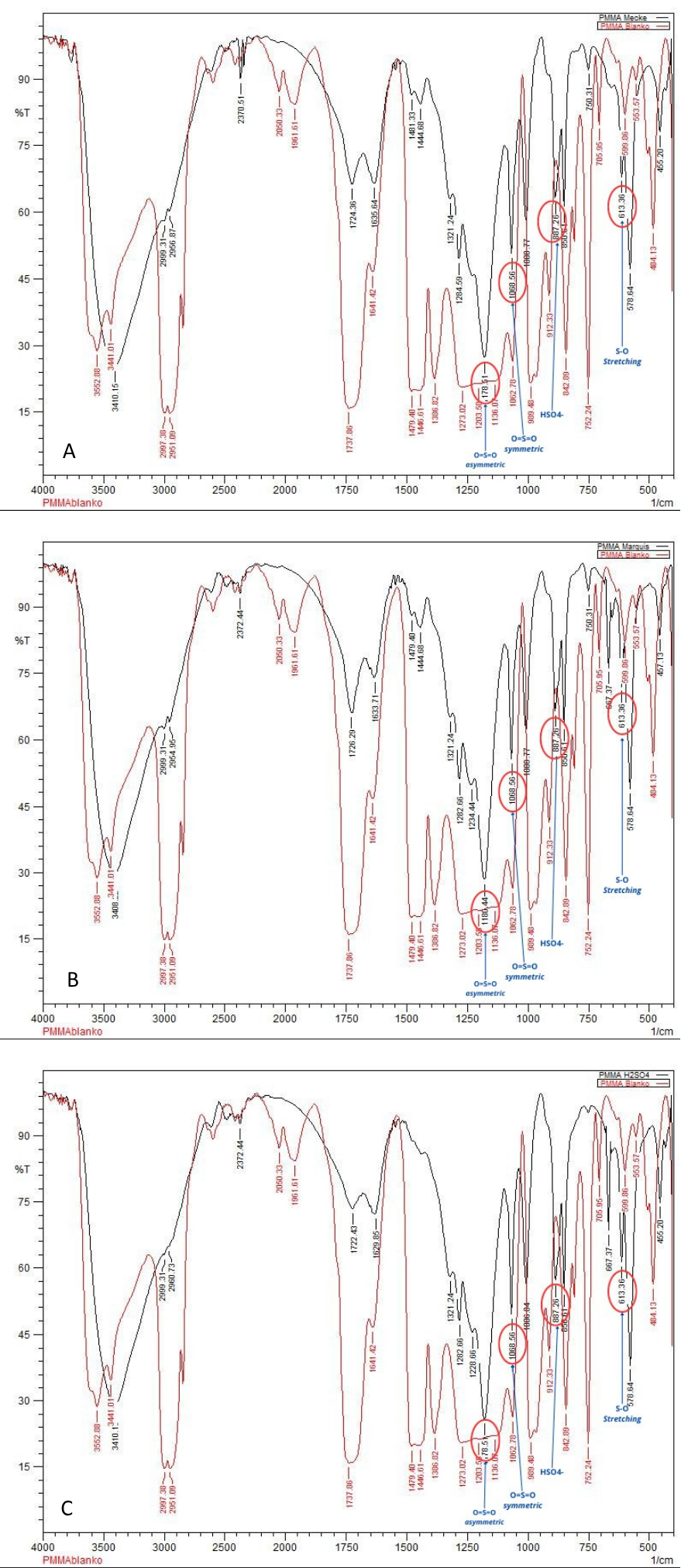


**Figure S1.** Comparison of the Infrared Spectrum of (A) PMMA and the Spectrum of PMMA Mixed with Mecke's Reagent, (B) PMMA and the Spectrum of PMMA Mixed with Marquis Reagent, (C) PMMA with the Spectrum of PMMA Mixed with Concentrated H_2_SO_4_ Reagent

**Table S3.** Selectivity Test Results of Indicator strips Against Tetracycline

| **Active substance** | **Mecke** | **Marquis** | **H_2_SO_4_** |
| --- | --- | --- | --- |
| Negative Control | 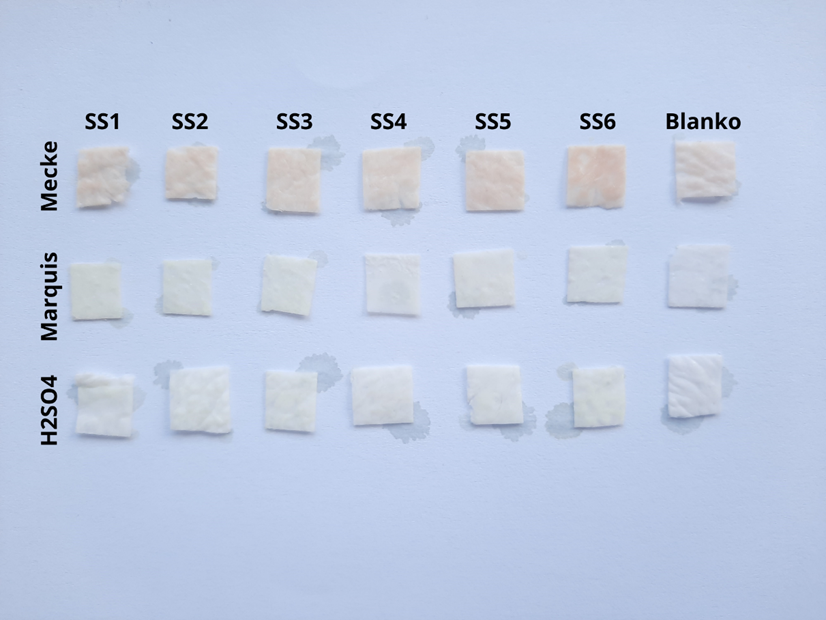 | 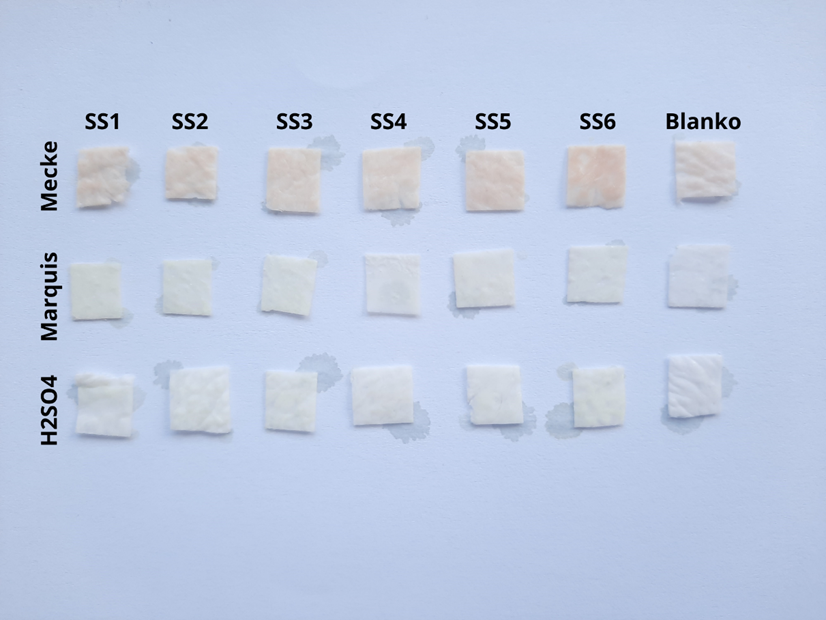 | 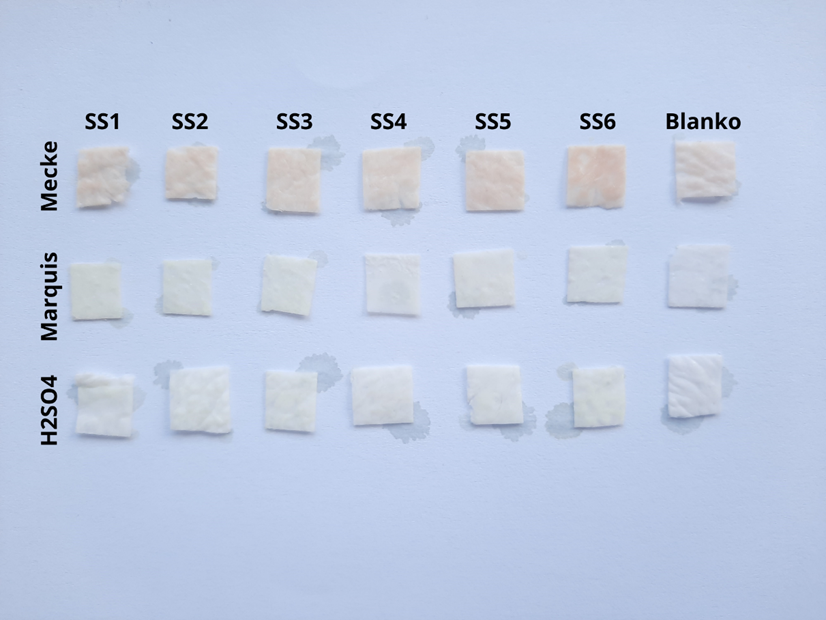 |
| Tetracycline | *Purple Red* (+)  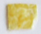 | Oranye (+)  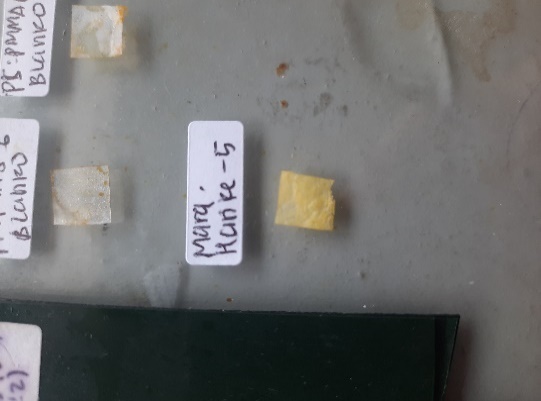 | Kuning (+)  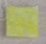 |
| Ampicillin | No color change (-)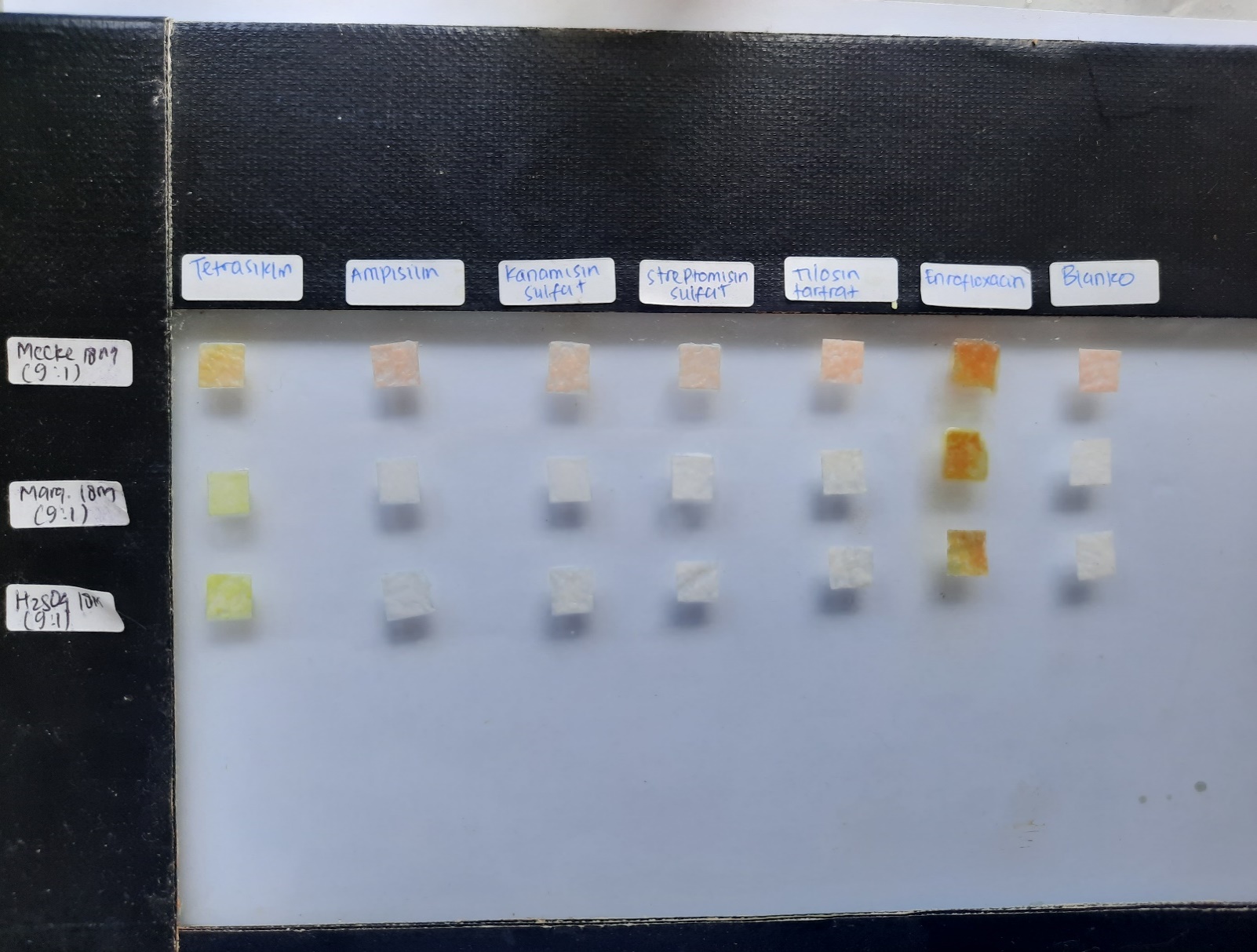 | No color change (-)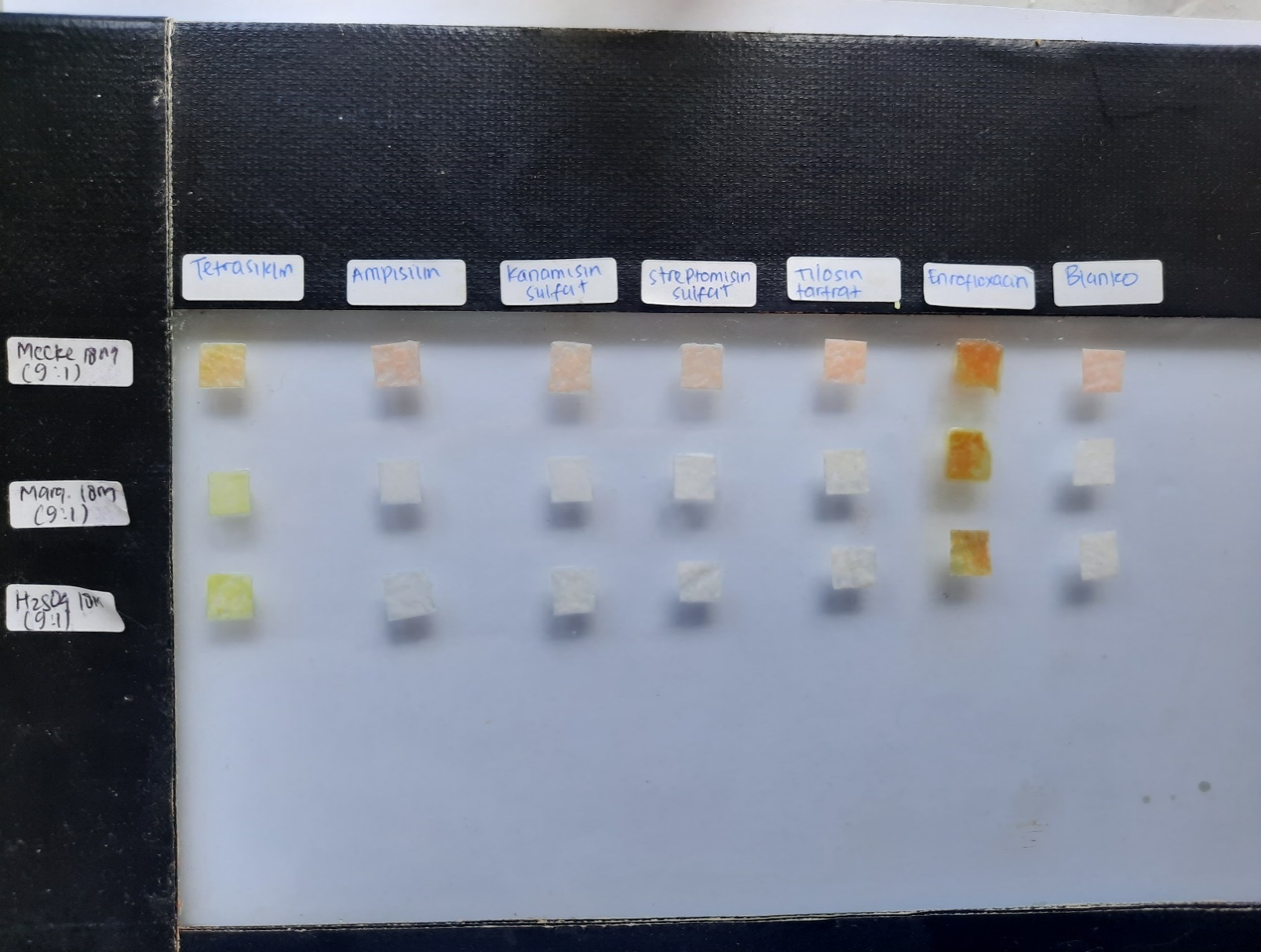 | No color change (-)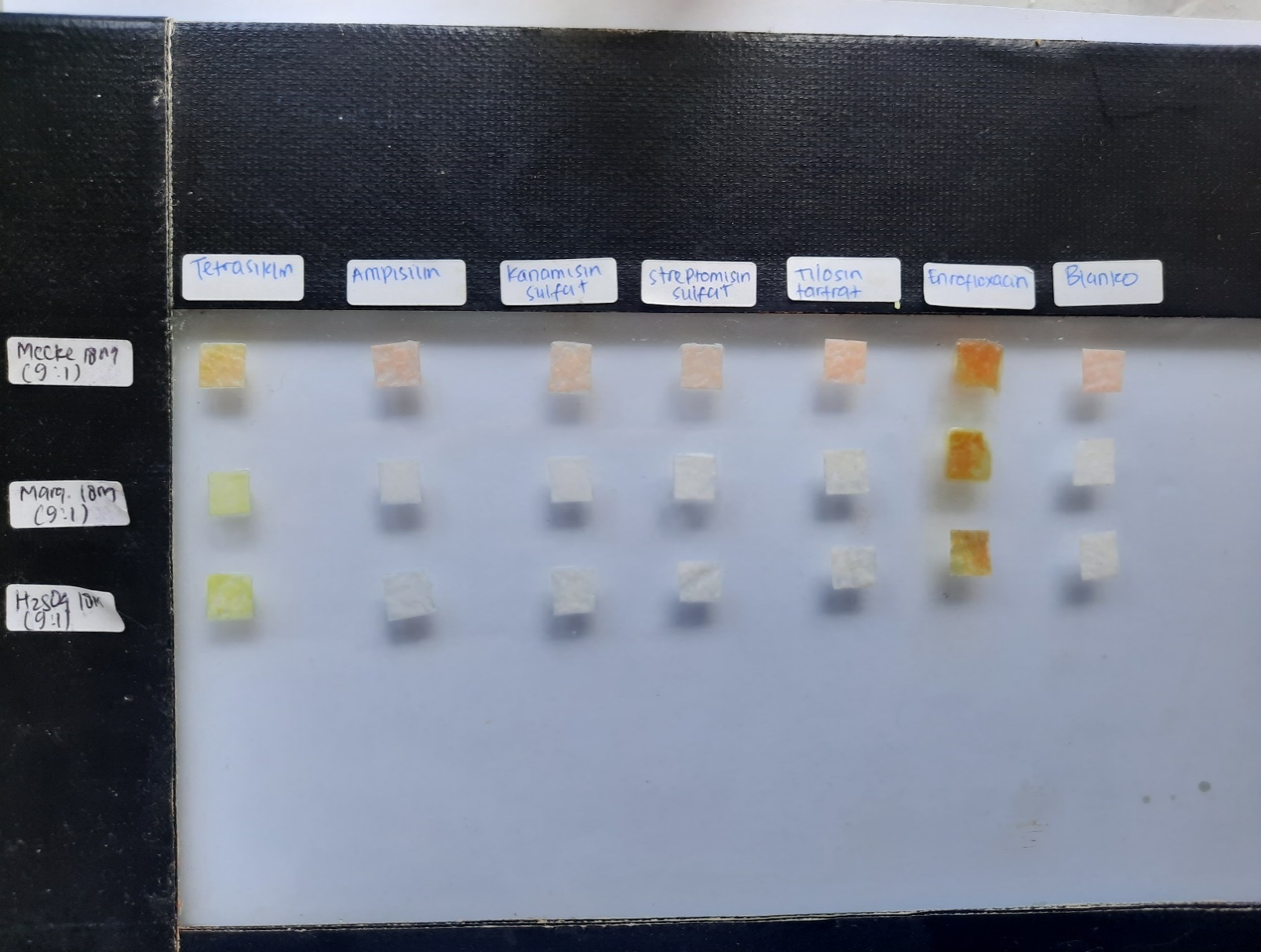 |
| Kanamycin sulfate | No color change (-)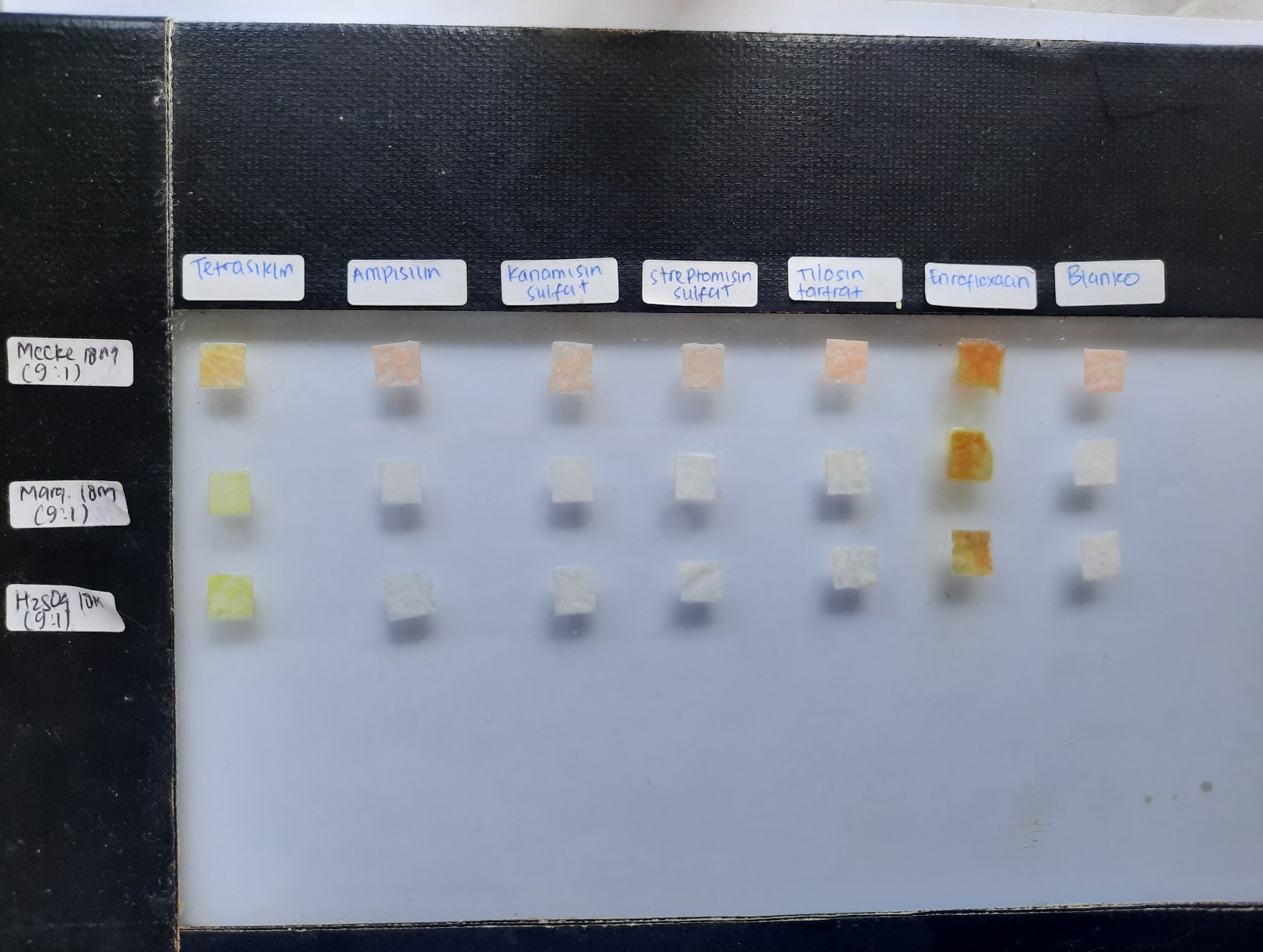 | No color change (-)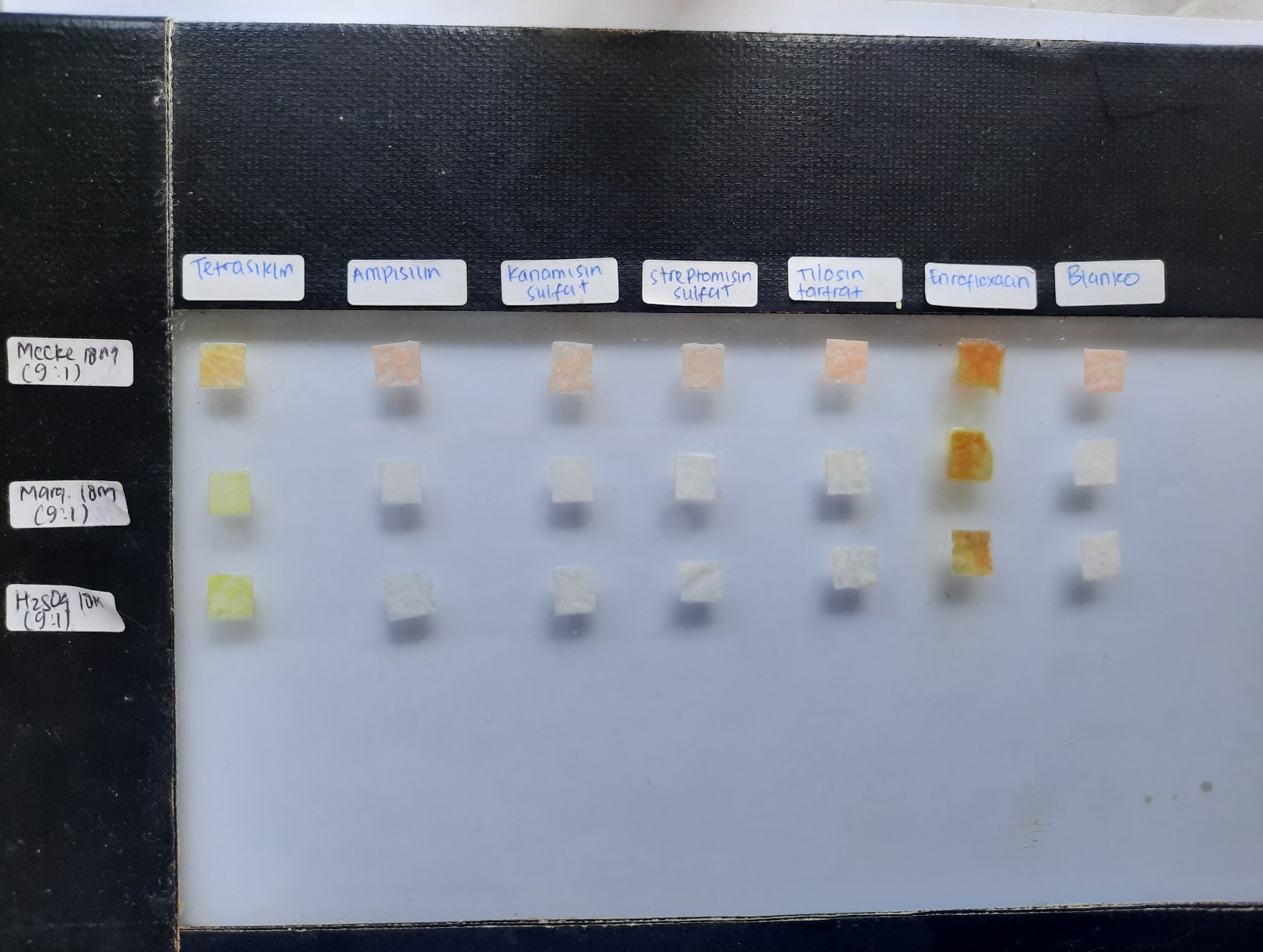 | No color change (-)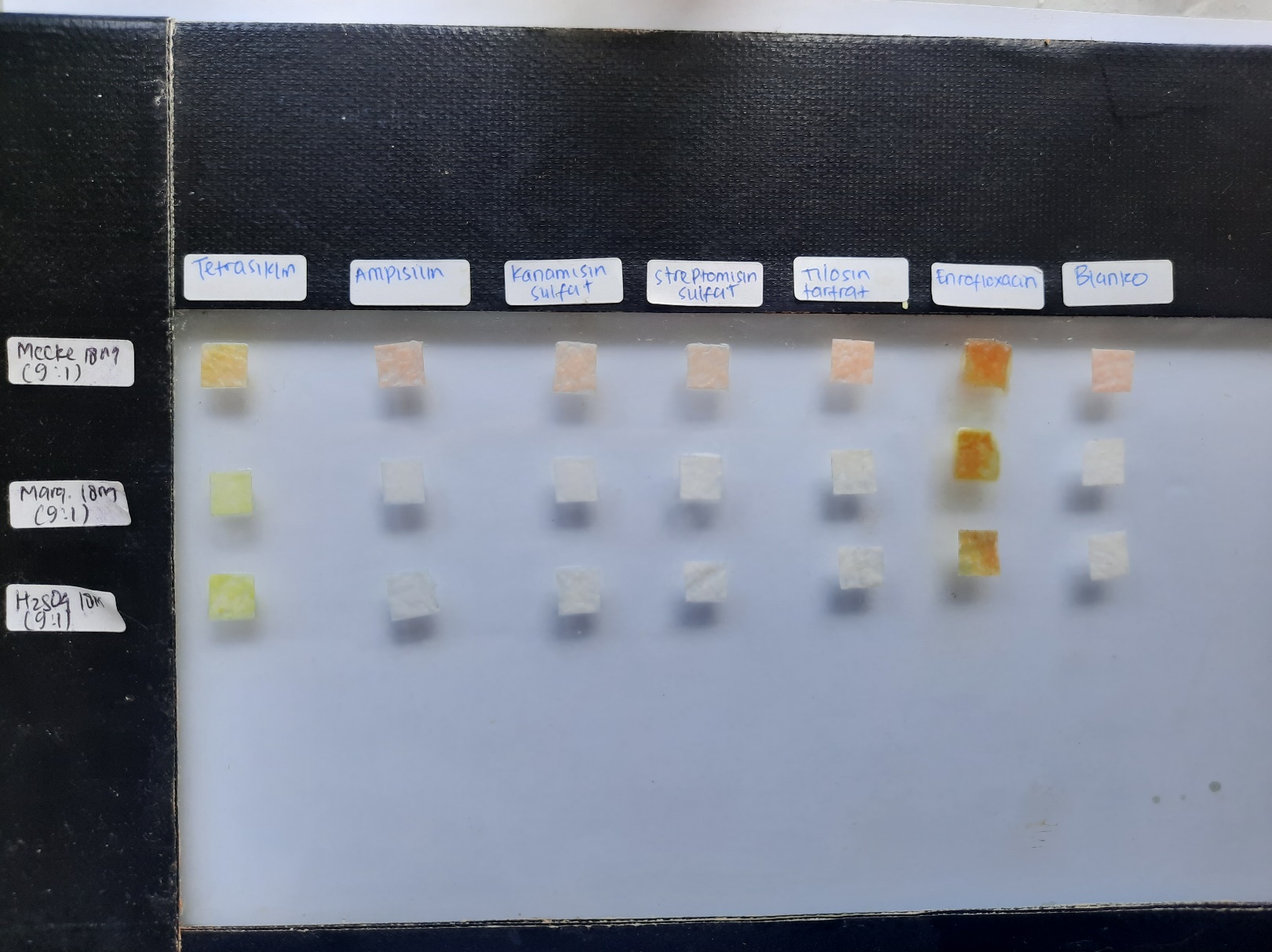 |
| Streptomycin sulfate | No color change (-)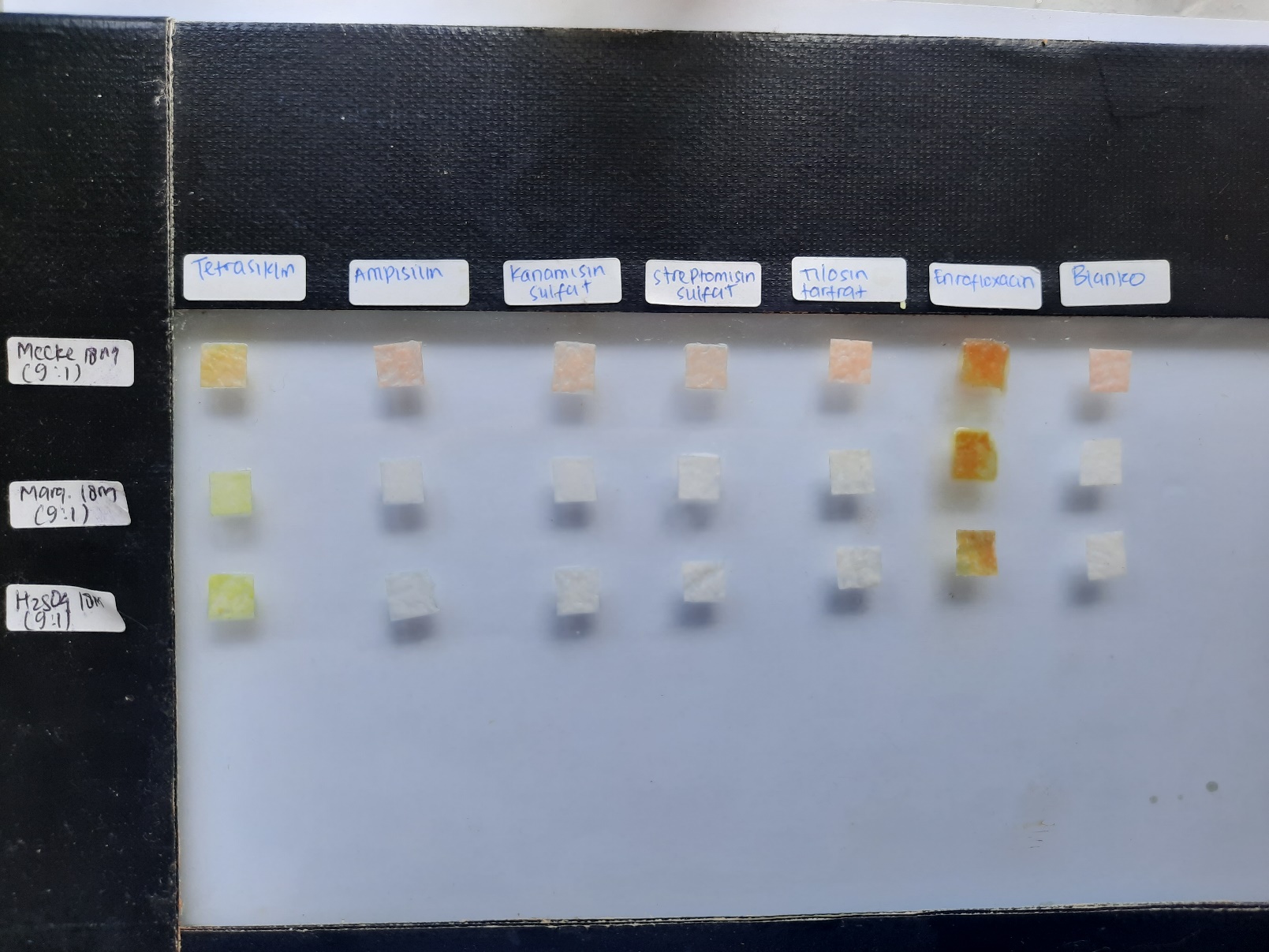 | No color change (-)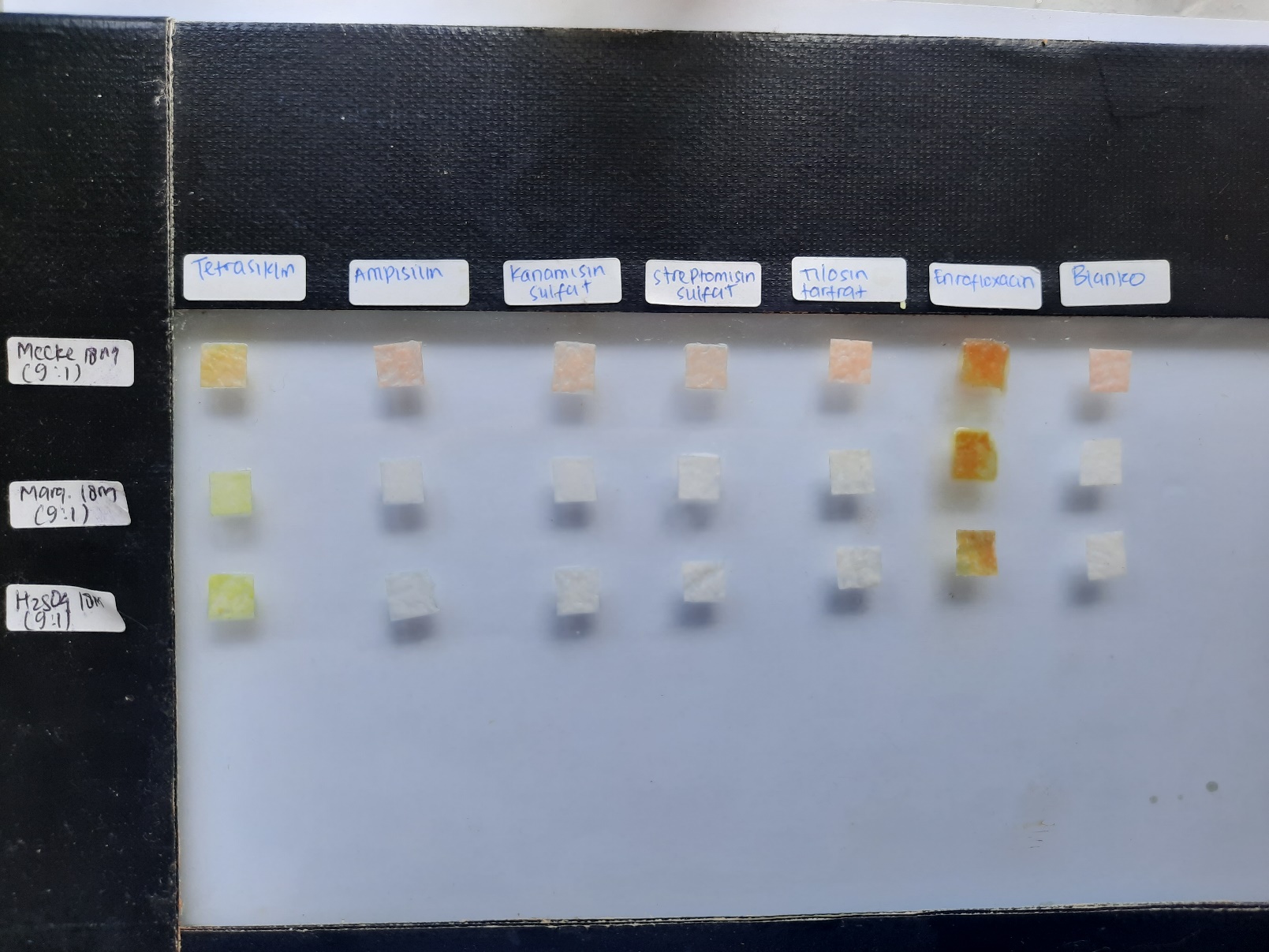 | No color change (-)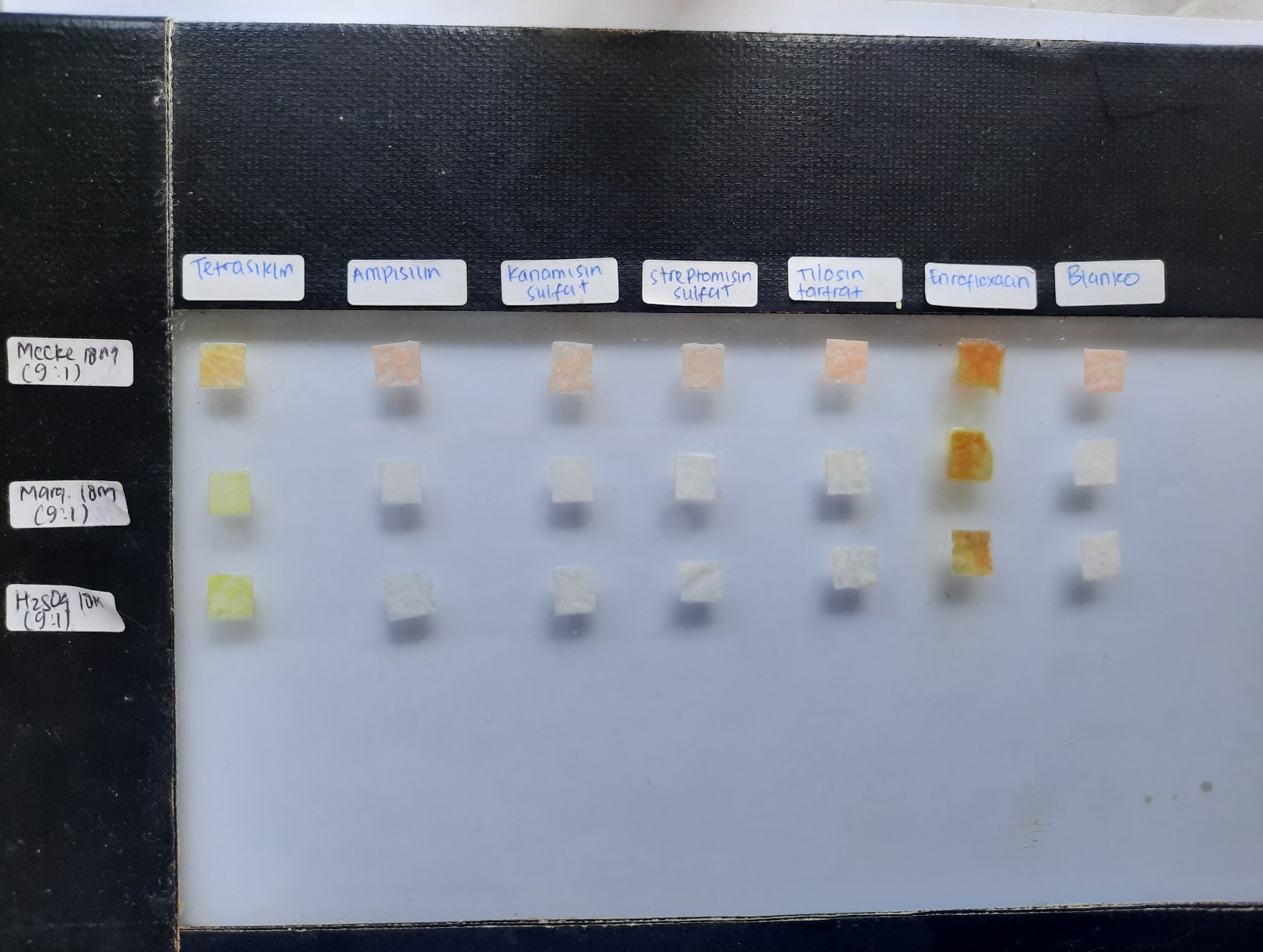 |
| Tylosin tartrate | Brownish black (-)  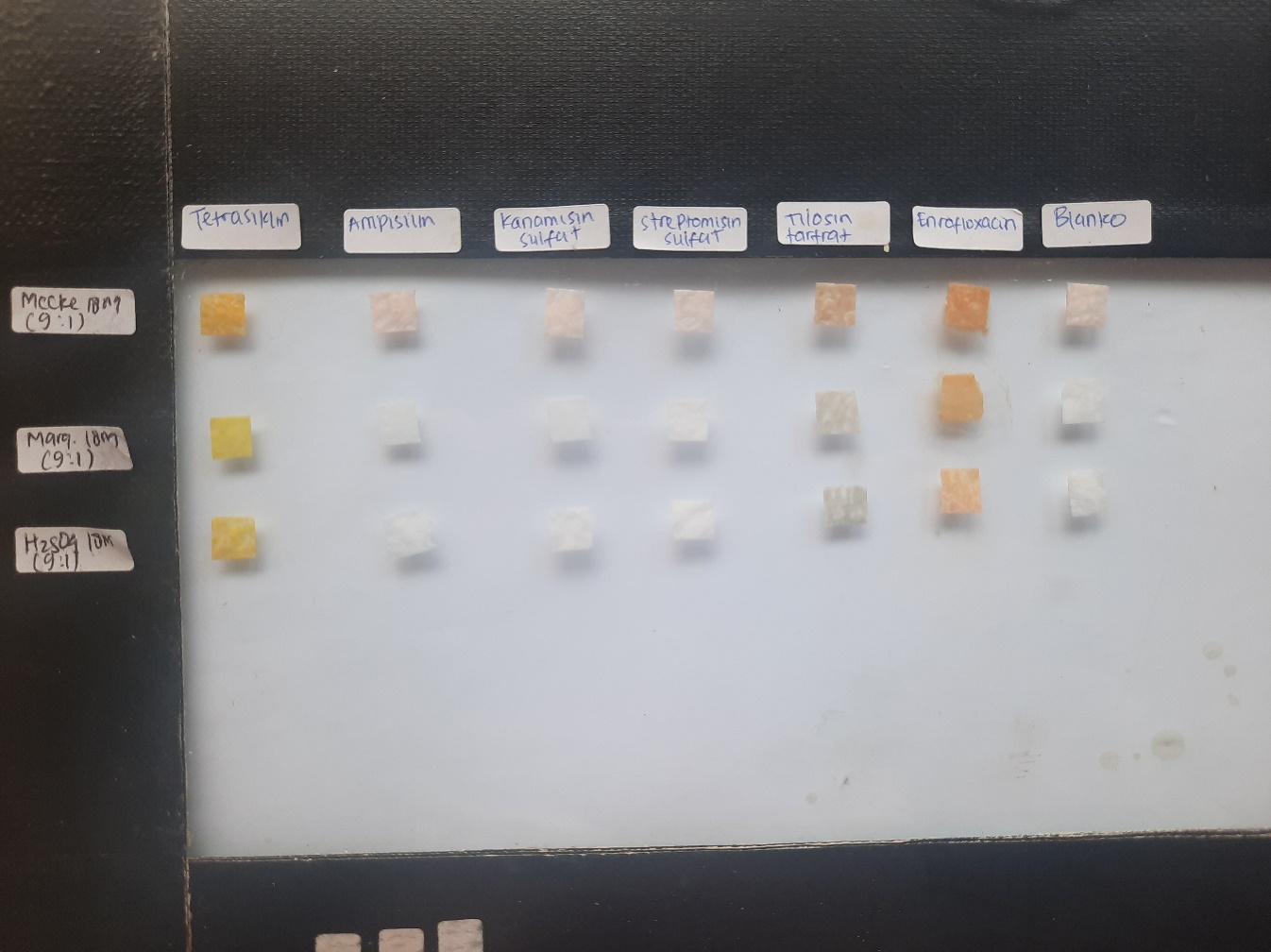 | Yellow-Brown (-)  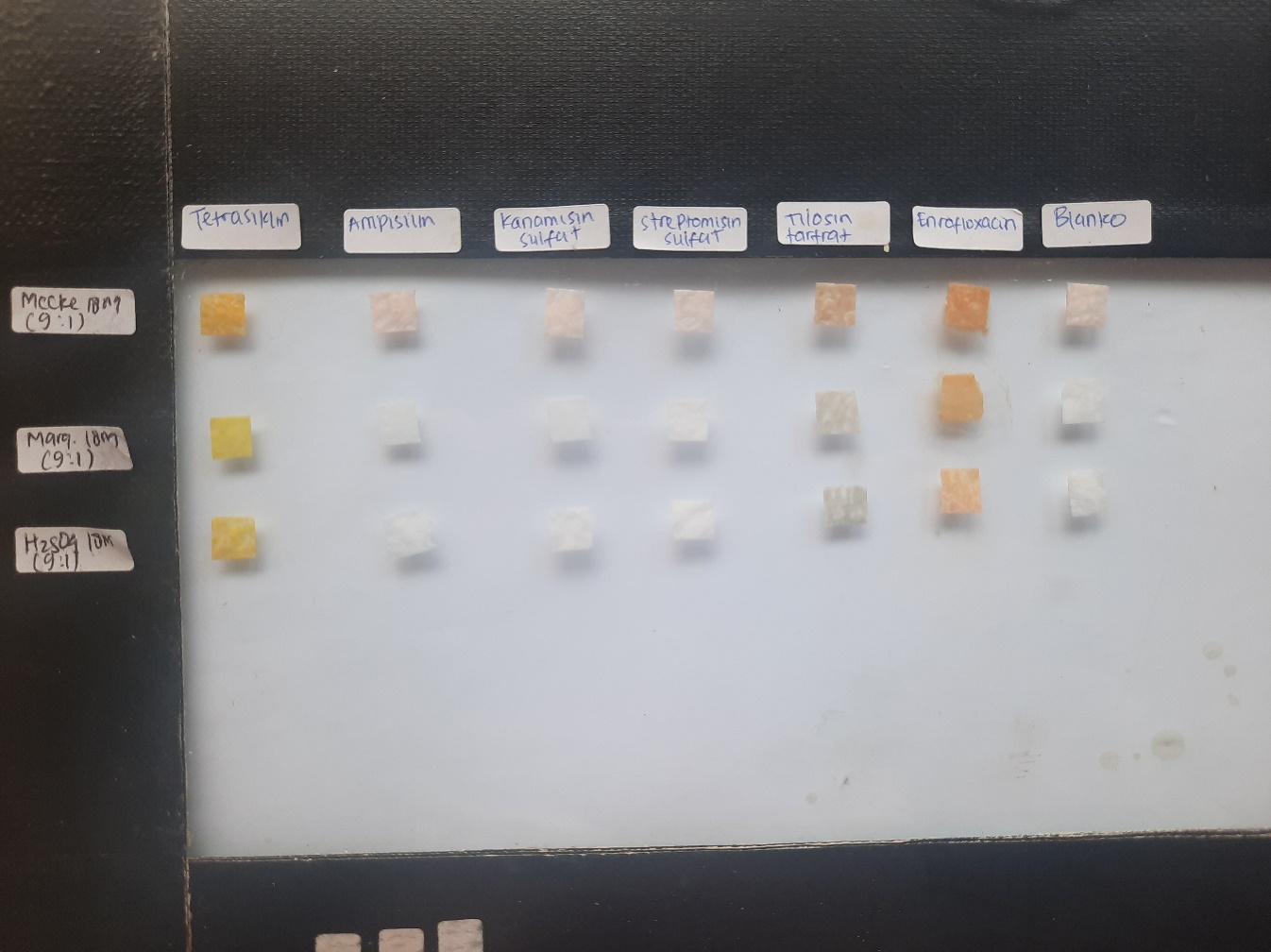 | Brownish black (-)  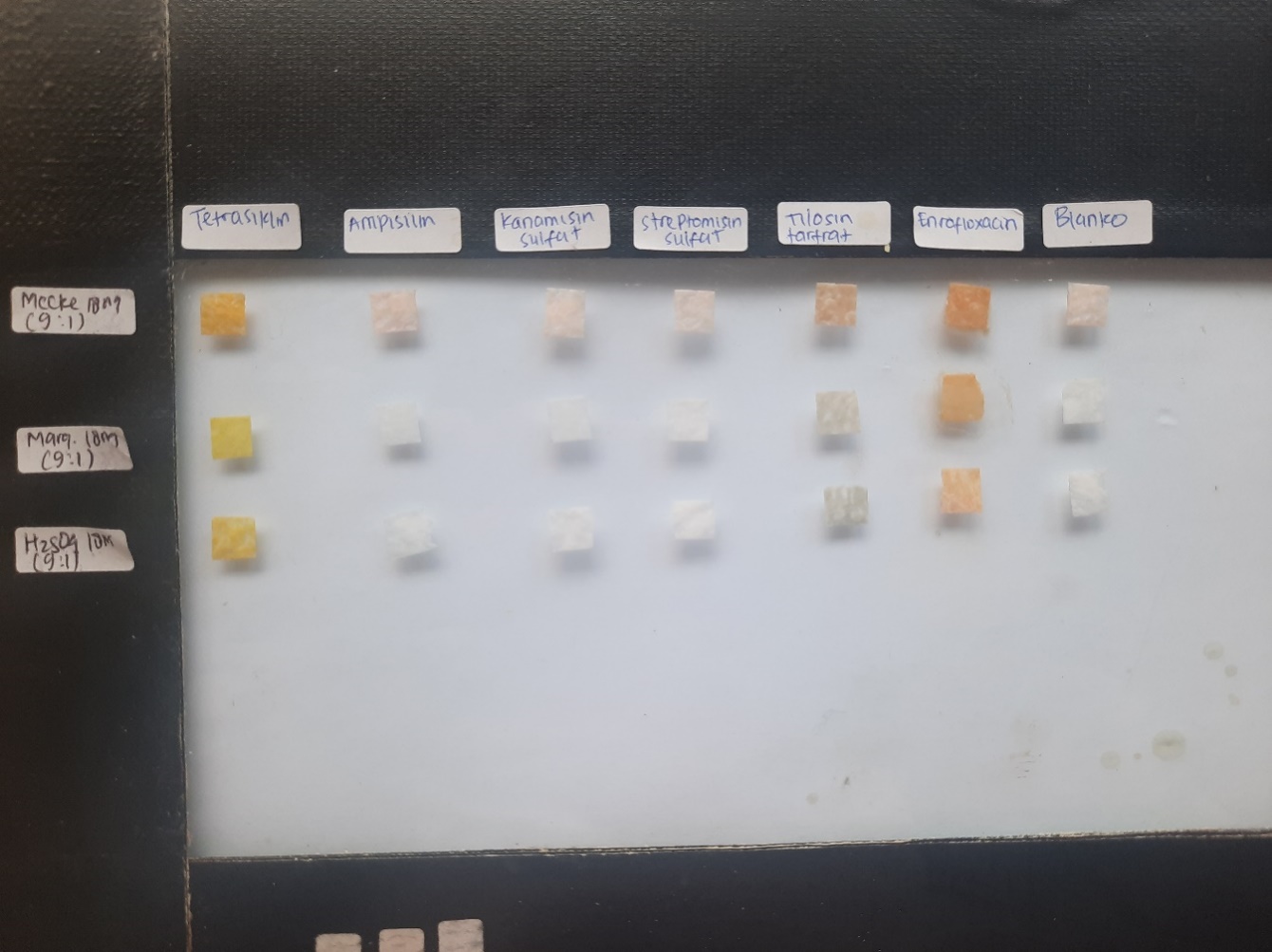 |
| Enrofloxacin | Brown (-)  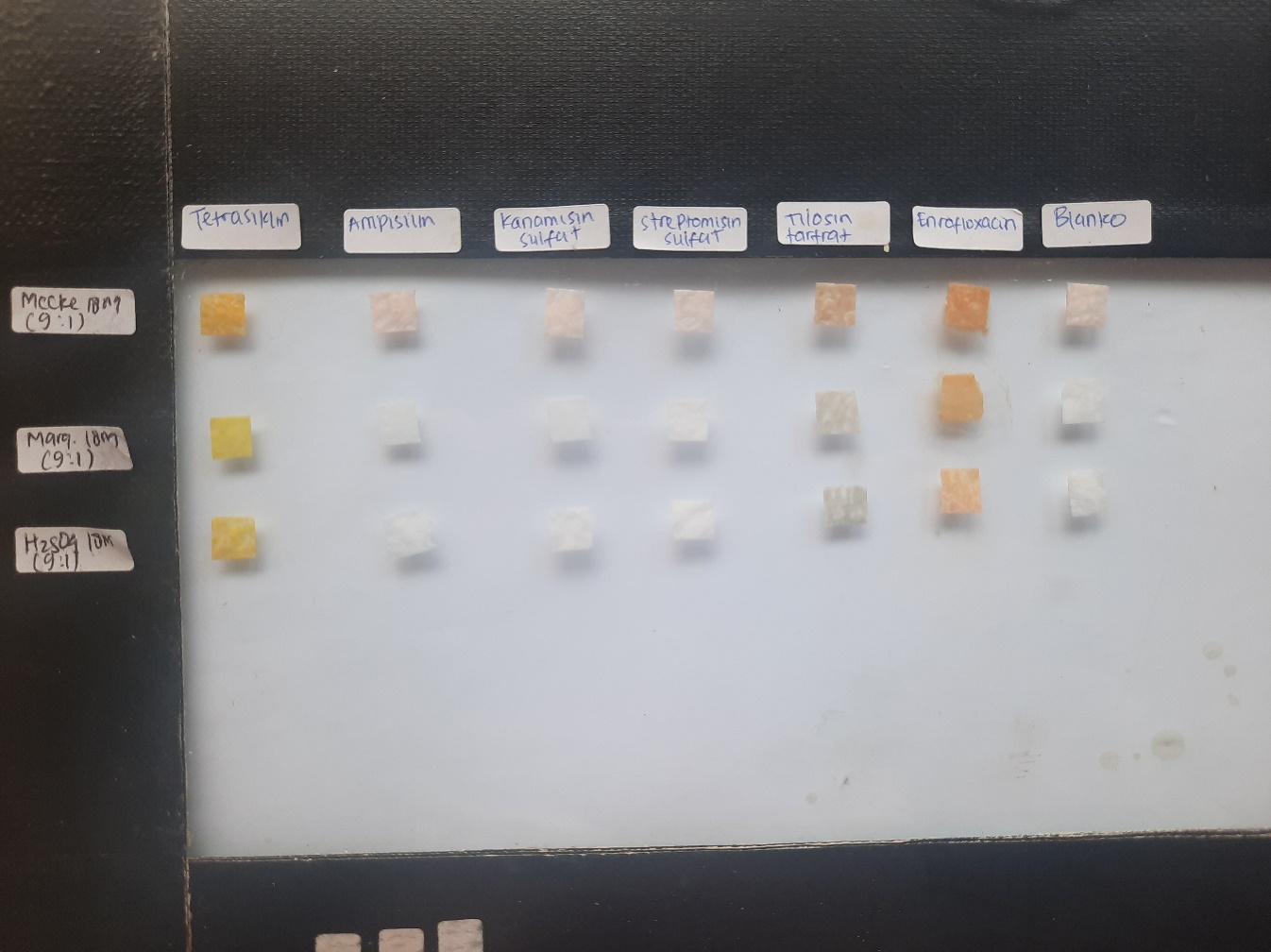 | Brown (-)  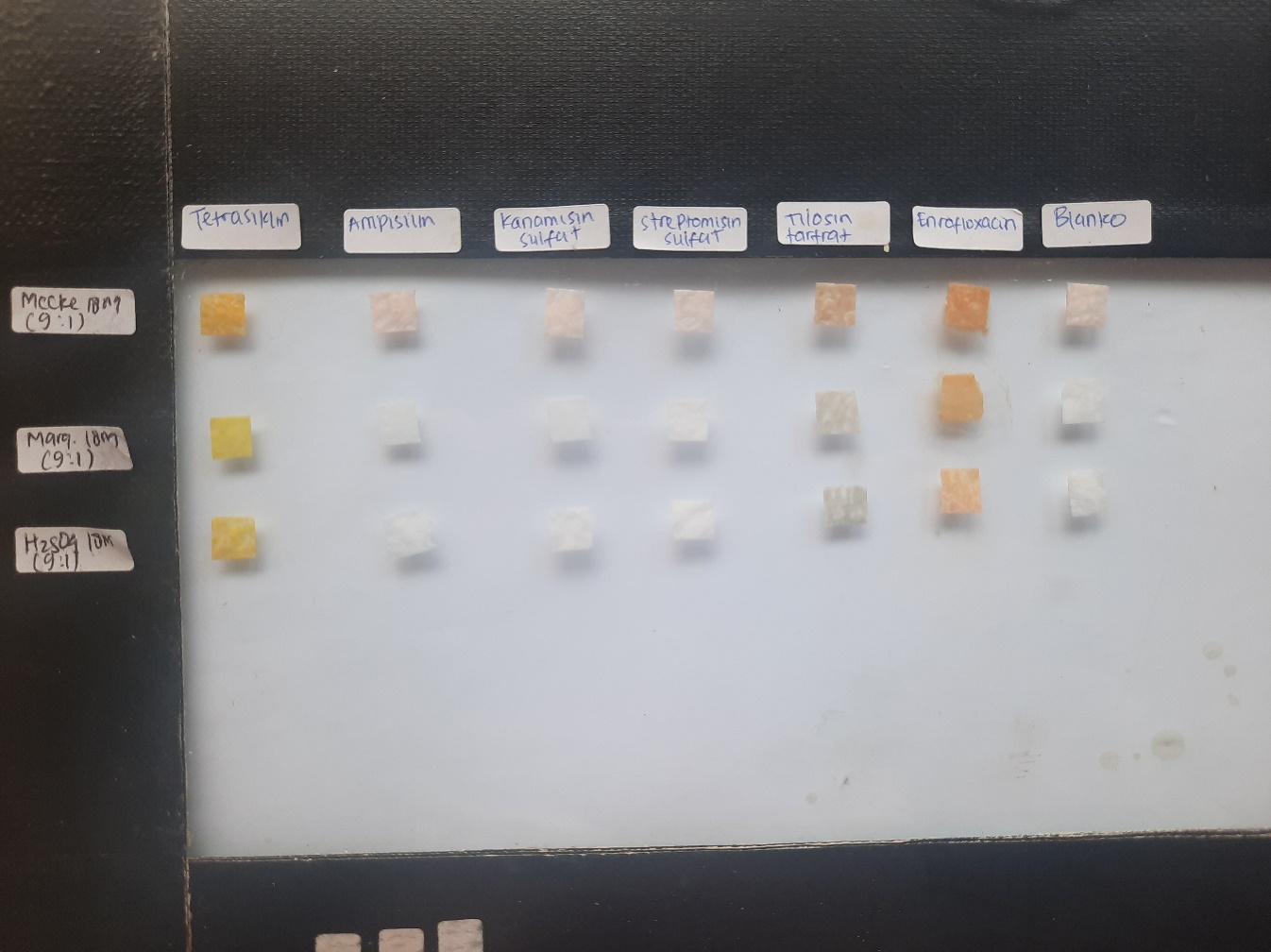 | Brown (-)  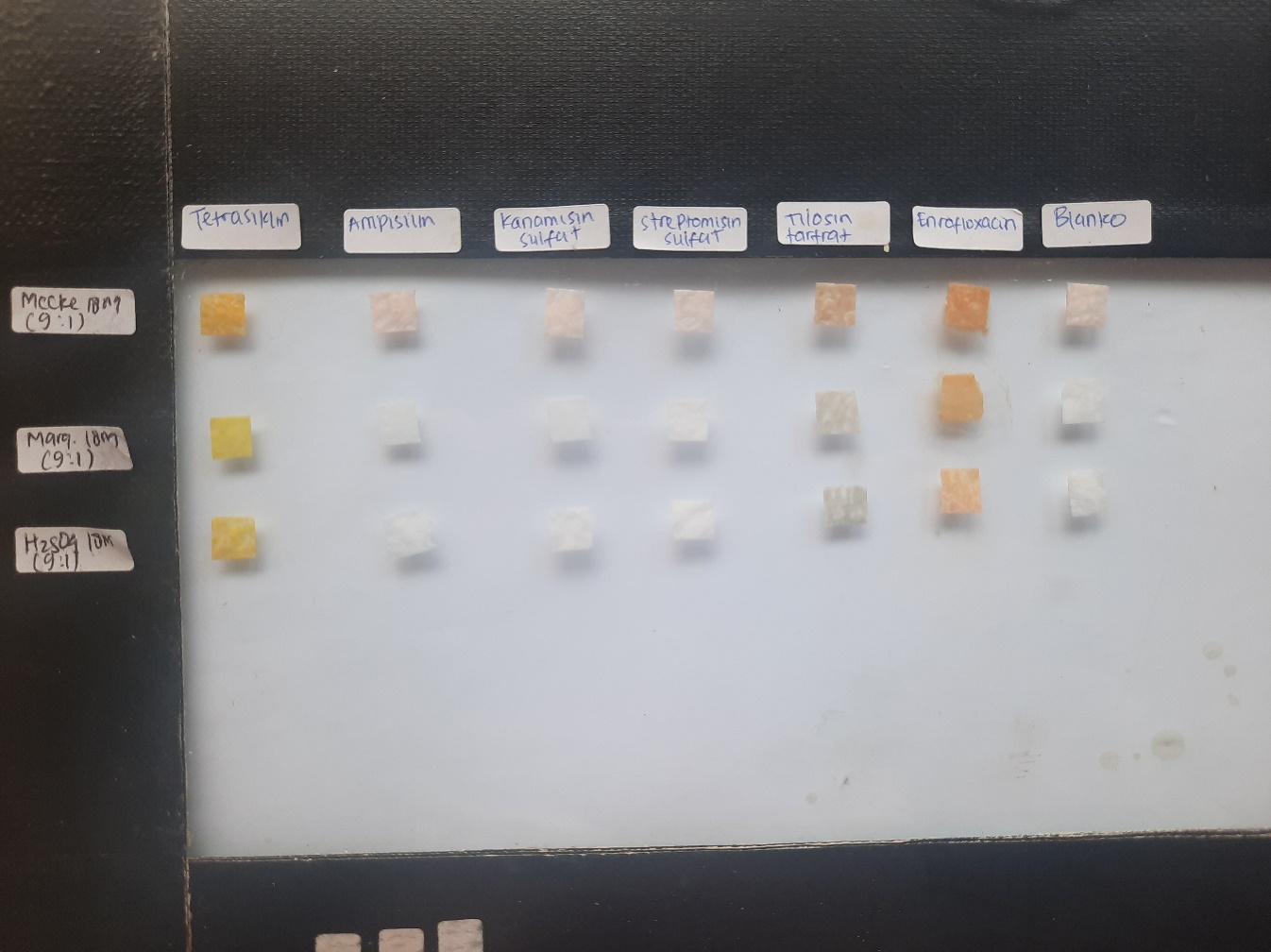 |

Information: (−) : Tetracycline not detected; (+) : Tetracycline detected

**Table S4.** Comparison of Rf Values ​​of TLC Screening Results of Tetracycline Standards

and Milk Samples

| **Sample Code** | **Rf Value** | **Result** |
| --- | --- | --- |
| T | 0.176 | + |
| S1 | - | - |
| S2 | - | - |
| S3 | - | - |
| S4 | - | - |
| S5 | - | - |
| S6 | - | - |
| SS1 | 0.176 | + |
| SS2 | 0.2 | + |
| SS3 | 0.21 | + |
| SS4 | 0.188 | + |
| SS5 | 0.176 | + |
| SS6 | 0.188 | + |

Information: T: Standard Tetracycline; S1, S2, S3, S4, S5, S6: Non Spike Sample; SS1, SS2, SS3, SS4, SS5, SS6: Spike Sample; (−): No tetracycline detected; (+): Tetracycline detected

**Table S5.** Results of Measurement of Tetracycline Residue Levels in Spiked and Unspiked

Milk Samples Using LC-MS/MS

| **Sample Code** | **Tetracycline Concentration (ppm)** |
| --- | --- |
| S1 | − |
| SS1 | 8.37 |
| S2 | − |
| SS2 | 7.38 |
| S3 | <0.022 |
| SS3 | 10.20 |
| S4 | 0.031 |
| SS4 | 9.06 |
| S5 | <0.022 |
| SS5 | 8.42 |
| S6 | − |
| SS6 | 9.68 |

Information : S1, S2, S3, S4, S5, S6: Non Spike Sample; SS1, SS2, SS3, SS4, SS5, SS6: Spike Sample; (−): No tetracycline detected
